# Preparation of a Growth Hormone Receptor/Prolactin Receptor Bispecific Antibody Antagonist Which Exhibited Anti-Cancer Activity

**DOI:** 10.3389/fphar.2020.598423

**Published:** 2020-12-10

**Authors:** Xin Chen, Di Wu, Yan Zheng, Xingxing Liu, Jianmeng Wang

**Affiliations:** ^1^ Department of Radiology, The First Hospital of Jilin University, Changchun, China; ^2^ Department of Breast Surgery, The First Hospital of Jilin University, Changchun, China; ^3^ Department of Geriatrics, The First Hospital of Jilin University, Changchun, China

**Keywords:** prolactin receptor, growth hormone receptor, breast cancer, antagonist, anti-idiotypic antibody

## Abstract

Prolactin receptor (PRLR) and growth hormone receptor (GHR) are closely related to the occurrence and development of breast cancer, and breast cancer cell endogenously express GHR, PRLR and GHR-PRLR heterodimer. In this case, the combined use of PRLR or GHR inhibitors may produce better anti-breast cancer potential than PRLR or GHR inhibitors alone. In this case, it is necessary to develop the dual-function GHR/PRLR antagonists with anti-breast cancer potential. For this, we used hybridoma technology to generate an anti-idiotypic antibody (termed H53). We then used various techniques, including competitive ELISA, competitive receptor binding analysis, and indirect immunofluorescence assay to identify H53, and the results show that H53 behaves as a typical internal image anti-idiotypic antibody (Ab2β). Further experiments indicate that H53 is a dual-function inhibitor, which not only inhibited PRLR-mediated intracellular signaling, but also blocked GHR-mediated intracellular signaling in a dose-dependent manner. Furthermore, H53 could inhibit PRL/GH-driven cancer cell proliferation *in vivo* and *in vitro*. This study indicates that H53 exhibits potential biological activity against breast tumors, which implies that internal image anti-idiotypic antibodies may be a useful strategy for the development of PRLR/GHR dual-function antagonists for breast cancer therapy.

## Introduction

Prolactin (PRL) and growth hormone (GH) is secreted by the anterior pituitary gland. PRL is structurally and functionally similar to growth hormone (GH) and placental lactogens (PL). Previous work indicates that the PRL, GH, and PL genes evolved from the same ancestral gene (Moriyama et al., 2006). PRL has a wide range of biological functions and is involved in almost all physiologic processes, including reproduction, water and electrolyte balance, growth and development, immune function, cell proliferation and differentiation as well as endocrine and metabolic functions (Moriyama et al., 2006). Based on published literature, both GH and PRL may have as many as 300 biological effects. Prolactin receptor (PRLR) and GHR comprise of an extracellular domain (ECD), transmembrane domain (TD), and intracellular domain (ID) (Ben-Jonathan et al., 1996). PRL and GH exert most of its biological functions through GHR/PRLR-activated intracellular signaling pathways, including JAK-STAT, and MAPK-ERK1/2 signal pathways among others (Chilton and Hewetson, 2005).

Dysregulated PRL/PRLR or GH/GHR activity has been associated with the development of various cancers, including breast cancer (Wagner and Rui, 2008; Goffin and Touraine, 2015; Xu et al., 2013). Globally, breast cancer is the leading cause of cancer deaths among women (Wagner and Rui, 2008; Goffin and Touraine, 2015). Multiple studies suggest that PRL and GH promotes breast cancer growth (Tworoger et al., 2006; Bernichtein et al., 2010; Conway-Campbell et al., 2007), leading to wide interest in PRLR/GHR as a potential target for breast cancer therapy (Damiano et al., 2013). To date, multiple PRLR and GHR antagonists have been developed. These fall into two categories: PRL/GH analogs and anti-PRLR/anti-GHR antibodies. PRL/GH analog inhibitors, such as G129R and G120R (Tallet et al., 2008; Goffin et al., 2005), are created by mutating one of the PRL/GH receptor-binding sites, which in turn block the biological effects of PRL/GH. In addition, Anti-PRLR/anti-GHR antibodies may block the interaction of PRL/PRLR and GH/GHR (Damiano et al., 2013). Although a series of GHR/PRLR inhibitors have been developed, but to date, there is still no GHR/PRLR inhibitors that can be used clinically.

Studies have indicated that PRLR and GHR are closely related to the occurrence and development of breast cancer, and breast cancer cell endogenously express GHR, PRLR (Xu et al., 2013). In this case, it is necessary to prepare a GHR-PRLR dual-acting antagonist. Although previous studies have found that G120R can be used as a dual-effect inhibitor against PRLR/GHR, a series of studies have shown that G120R itself may be a weak activator (Langenheim et al., 2006; Clevenger et al., 2008).

In this study, the anti-idiotypic antibody strategy was used to prepare GHR/PRLR antagonists. According to Jene’s immune network theory (Mulay et al., 1974), the variable region of an antibody (Ab1) produced by antigen, can in turn be used as an antigen to produce anti-antibodies (Ab2), termed anti-idiotype antibodies. Anti-idiotypic antibodies fall into four classes, Ab2α, Ab2γ, Ab2ε and Ab2β. Of these, type Ab2β structurally mimics the initial antigen, and is termed as an “internal image” of the initial antigen (Beck et al., 2002). We prepared the GHR/PRLR dual-effect antagonist (H53) by anti-idiotypic antibody strategy, and found that H53 exhibits potential effects against breast tumors.

## Materials and Methods

### Materials

Anti-total JAK2, anti-total STAT5/3, anti-total ERK1/2, anti-total AKT, anti-phospho-specific JAK2, anti-phospho-specific STAT5/3 and anti-phospho-specific AKT antibodies were purchased from Cell Signaling Technology (United States). Extracellular domain of PRLR (termed as PRLR-ECD) was purchased from Hua-Yi Biological Inc. (Changchun, China). Cell Counting Kit-8 was purchased from Genview (Shanghai, China). Cell culture medium and fetal calf serum (FCS) were obtained from Gibco (United States). BSA, PVDF membrane and ECL were purchased from Beyotime Biotechnology (Shanghai, China). ELISA plates and cell culture plates were obtained from Corning. HAT and HT were purchased from Invitrogen (California, United States). Skimmed milk, low-fluorescence PVDF membrane and ECL were purchased from Hua-Cheng (Changchun, China). HAT and HT were purchased from Invitrogen (California, United States). GHBP was purchased from Abcam. Prolactin receptor binding protein (PRLR-ECD) was obtained from Hua-Cheng Biotechnology Co., Ltd. Anti-PRLR was purchased from Abcam. Prolactin antagonist (des one to nine, G129R) was obtained from PROSPEC (CYT-1066). Unless otherwise specified, reagents were purchased from Sigma company.

### Cell Culture

Breast cancer cell line MCF-7 and T47D cells were purchased from ATCC (United States) and cultured in DMEM high glucose medium supplemented with 10% FBS, penicillin and streptomycin at 37°C in a humidified atmosphere of 5% CO_2_. In addition, CHO cells were also cultured in DMEM medium.

### Establishment of Growth Hormone Receptor/Prolactin Receptor Transfected Cell Lines

One day before transfection, CHO cells in the logarithmic growth phase were harvested and seeded in 6-well cell culture plates at 2 × 10^5^ cells per well. They were then cultured for 12 h at 37°C in DMEM/F12 supplemented with 10% FBS. When the cell reached 70%–90% confluence, culture media was replaced with serum-free Opti-MEM medium. Lipofectamine 3,000 was then mixed with pc-DNA3.1-PRLR/GHR recombinant plasmid, after which Lipofectamine 3,000/pc-DNA3.1-GHR/PRLR mix was then added into each well and incubated for 2–3 h. After transfection, the transfection medium was replaced with the DMEM/F12 supplemented with 10% FBS and cultured for 24 h. The cells were then harvested by trypsinization with 0.25% trypsin and transferred into a 100 mm Petri dish. They were then treated with G418 at 600 μg/ml. Control cells were transfected with empty vector. After two weeks, G418 resistant clones were transferred into 24-well plates for expansion. Stably transfected PRLR/GHR CHO cells were obtained by limiting dilution.

### Indirect ELISA for Ab1 and Ab2

To detect Ab1 by ELISA, microtiter plates were coated with 100 μL of 3.0 μg/ml PRL/GH per well and incubated at 4°C overnight. The plates were then rinsed thrice with PBST and blocked with 100 μL of 5% sheep serum for 2 h at 37°C. After washing, cell culture supernatant, or serum from an immunized animal was added into the microtiter plates. Serum collected before immunization was used as negative control, and PBS was used as a blank control. The plates were then incubated at 37°C for 1 h. After washing thrice, 100 μL of HRP-conjugated secondary antibody diluted at 1:5,000 was added into each well and incubated for 1 h. After washing, 50 μL TMB was added to each well for signal development, and the absorbance was measured at 450 nm using a microplate reader (Bio-rad).

In addition, to establish the ELISA system for detecting Ab2, the F (ab′)2 fragments of anti-G120R antibodies were coated on the microtiter plate, the experiment protocols are the same as above described for detection of Ab1.

### Anti-Idiotypic Antibodies (Ab2β) Screening

GH can bind both GHR and PRLR, and GH binds GHR and PRLR through its receptor-binding site 1 (GH contains two receptor-binding sites, termed as Site1 and Site2, respectively). In addition, GH’s PRLR-binding site is overlapped with that of PRL’s PRLR-binding site (Mulay et al., 1974; Chilton and Hewetson, 2005; Langenheim et al., 2006); therefore, GH also can inhibit PRL from binding to PRLR (Chilton and Hewetson, 2005). Based on this, G120R [G120R is an analogue of GH, but it contains only one receptor binding site (Site1)] was used as an antigen to develop the dual-function GHR/PRLR antagonist through anti-idiotypic antibodies strategy, and anti-idiotypic antibody screening was performed as previously reported (Mulay et al., 1974; Langenheim et al., 2006). The screening consisted of the following steps (the specific anti-idiotypic antibody screening strategy is shown in Supplementary Figure S1): 1) preparation of anti-GH antibodies (Ab1), 2) preparation of monoclonal anti-idiotypic antibody to GH (Ab2), 3) screening for anti-idiotypic antibodies that could bind PRLR and GHR, 4) identification of anti-idiotypic antibodies that are antagonistic to GHR/PRLR (Ab2β).

### Production of anti-GH Antibodies

G120R was dissolved in normal saline and emulsified in equal volume of Freund’s complete adjuvant, animal immunization procedures are as follows: 1) for the first immunization, New Zealand white rabbits weighing 2.5 kg, were injected with 0.8 mg of G120R. At 14 days intervals, the rabbits were injected with G120R emulsified with incomplete adjuvant. Three days after the final injection, serum antibody titer (anti-G120R) was measured by ELISA assays. After the serum titer had met the experimental requirements, blood was collected and IgG fragments were purified by protein-A affinity chromatography.

### Preparation of Monoclonal Anti-Idiotypic Antibodies to GH (G120R)

Six BALB/c mice were immunized with anti-G120R antibody [F(ab′)2 fragments] on the first, seventh, and 14th days. The first immunization was done using 50 μg of Ab1 mixed with Freund’s complete adjuvant. In the second and third immunizations, the mice were injected with anti-G120R antibody’s F(ab′)2 fragments mixed with Freund’s incomplete adjuvant. After the final immunization, the blood from tail vein of the immunized mice was taken to determine antibody titer. When the titer exceeded 1:20,000, spleen cells from the immunized mice were fused with Sp20 according to the lan’s method (Hainan et al., 2017), and ELISA was used to detect positive clones. Five BALB/c mice were immunized with normal rabbit serum IgG as negative control.

### Screening for Ab2β

Two competitive ELISA assays were used to screen internal anti-idiotypic antibodies. 100 μL of 1 μg/ml GH was added into the wells of a microtiter plate and incubated for 1 h at 37°C. The plate was then washed three times with PBST and blocked with 3% BSA at 37°C for 2 h Ab1 and increasing concentrations of Ab_2_’s F(ab)_2_ or F(ab)_2_ of the control antibody were added. After three washes with PBST, 100 μL of IgG (Fc-specific)-HRP secondary antibody was added and incubated for 1 h. After three washes with PBST, 50 μL of TMB was added for signal development. Next, the F(ab)_2_ of anti-GH at 2 μg/well was used to coat microtiter plate for 1 h. After washing, the constant Ab1 mixed with increasing concentration of GH were added, respectively. Subsequent experiments were done as described above.

### Evaluation of Ab2β (H53)

To assess if H53’s receptor-binding epitope is overlapped with that of ligands (GH and PRL), we carried out competitive receptor-binding assays as previously described (Mulay et al., 1974; Langenheim et al., 2006). In brief, CHO-PRLR or CHO-GHR cells were serum starved for 8 h. The cells were then harvested and dispersed in a 24-well cell culture plates. Next, fluorescent labelled-PRL/GH and various concentrations of unlabeled PRL/GH, Ab2β or isotype-matched control antibodies were added and incubated at 4°C for 1 h. Cell sample were then analyzed using FACS Calibur (BD) to detect the fluorescence signal.

### Immunoprecipitation

Cells were collected and lyzed with 1 ml cell lysis buffer. They were then homogenized by gentle shaking at 4°C for 1 h. The cell lysates were then centrifuged at 12,000 rpm for 15 min and the supernatant collected. Control IgG and 30 μL of protein A agarose were added and mixed at 4°C for 1 h. The samples were then centrifuged at 12,000 rpm for 20 s, after which, the indicated antibodies were then added and mixed at 4°C for 1 h. Next, 50 μL of protein A agarose was added and the samples were incubated overnight at 4°C. The samples were then centrifuged for 20 s at 12,000 rpm. They were then washed 4 times, and 2X SDS loading buffer was added. The samples were then denatured at 99°C for 10 min followed by Western blot analysis.

### Western-Blot

The protein of the cells was extracted with a total protein extraction kit. Then, 30 μg of protein was subjected to 10% SDS-PAGE and transferred to a low-fluorescence PVDF membrane. The membranes were blocked with a 5% skimmed milk powder blocking solution at 37°C for 2 h. After washing, the membranes were incubated with the indicated primary antibody for 2 h. After washing three times, fluorescently labeled secondary antibody (1:5,000 dilution) were added at 4°C overnight. After washing the membrane 3 times with TBST, it was detected using a fluorescence system (Bio-Rad).

### Immunoelectron Microscopy

The cells were collected and washed for three times, the cells were then fixed in a 2% PFA + 0.25% glutaraldehyde solution for 15 min. The cells were then washed and dehydrated using gradient alcohol. They were then embedded with Epon812 epoxy resin, polymerize at 37°C for 12 h, and polymerized at 45°C for 60 h. Embedded samples were then sectioned and treated with 1% H_2_O_2_ for 30 min to expose the antigen. After washing with double distilled water, the sections were blocked with 1% BSA at room temperature for 1 h. Next, they were incubated with primary antibodies (control antibody and commercial GHR/PRLR antibody) and for 1 h. They were then washed with TBS (pH 7.4) and blocked with 1% BSA for 1 h. The cells were then incubated with 5 and 10 nm colloidal gold labeled secondary antibodies and incubated at room temperature for 60 min. After washing, the samples were examined using a transmission electron microscope (Hitachi H-7650)**.**

### Laser Confocal Microscopy Observation

Cells grown on glass slides were fixed in 4% PFA for 30 min at room temperature. The cells were then washed and permeabilized with 0.1% triton X-100 for 15 min at room temperature. After washing, the cells were blocked with 3% BSA for 60 min and then incubated with the indicated primary antibodies for 1 h. After washing, the cells were incubated with fluorescently labeled secondary antibodies for 1 h. After washing, the cell nuclei was counterstained with DAPI before the samples were examined by a confocal microscopy (Olympus FV3000).

### Native Gel Electrophoresis Analysis of Interaction of and Ab2β and Growth Hormone Receptor/Prolactin Receptor

To evaluate interaction between PRLR/GHR and Ab2β, non-denaturing electrophoresis was done. The extracellular domain of PRLR (PRLR-ECD) or GHR (GHR-ECD) was labeled with 5-iodo-acetamido fluorescein (IAF) (Beck et al., 2002). For binding experiments between PRLR/Ab2β or GHR/Ab2β, native gel electrophoresis was done and the gels were imaged using Biorad’s ChemiDoc XRS Gel Imaging System.

### Cell Proliferation Assay

To further evaluate the antagonistic activity of H53, we performed cell proliferation analysis using CCK-8 kit following the manufacturer’s instructions. Briefly, 100 μL of 1 × 10^4^ T47D cells were seeded into each well of 96-well plate. Next, the mixture of constant H53 and an increasing concentration of H53 or an isotype-matched control antibody were added to the cells and incubated incubate at 37°C, 5% CO_2_ for 24 h. The CCK-8 assay was then done following the manufacturer’s protocol. Absorbance was read using a microplate reader at 450 nm.

### RT-qPCR

RNA was extracted using TRIZOL kit according to manufacturer’s instructions. cDNA was synthesized using the PrimeScript RT reagent Kit (TaKaRa Biotechnology) according to manufacturer’s instructions. Bcl-2 mRNA levels were assayed by RT-qPCR using the following primers: Fwd: 5′-CAC​TCA​GCA​TAT​GGC​GCA​CGC​TGG​GAG​AAC​GGG​GTA​C‐3′; Rvs: 5′-GCG​AAG​CTC​TCG​AGC​TAT​CAC​CGC​ATG​CTG​GGG​CCG​TAC​AGT​TC‐3′). *β*-actin was used as the reference gene, and no template control was set. The experiment was done in triplicate.

### H53 Affinity Determination by Surface Plasmon Resonance

The extracellular domain of GHR and PRLR was coupled to a GLC chip via 1-Ethyl-3-(3-imethylaminopropyl) carbodiimide Hydrochloride and N-hydroxysulfosuccinimide (sulfo-NHS), and then injected with different concentrations of H53. The dissociation constant (Kd) was analyzed by the ProteOn XPR36 system.

### NIRF-Electrophoresis Mobility Shift Assay Analysis

The cells were stimulated with PRL or a mixture of PRL and different concentrations of H53, GH or a mixture of GH and different concentrations of H53. The cells were then washed with ice cold PBS, and the nuclear protein of 3 × 10^6^ cells were extracted using a nuclear protein extraction kit. The nuclear protein concentration was the determined using a BCA assay kit. Fluorescently labeled DNA probe sequence was mixed with nucleoprotein from different treatments. The mixture was then subject to EMSA (electrophoresis mobility shift assay) (Martens et al., 2005). The gels were imaged on an ODYSSEY infrared gel imager (Li-Cor). For supershift analysis, nuclear protein extracts were incubated on ice for 40 min with 1.5 mg of monoclonal anti-Stat5.

### The Effect of H53 on the Clone Formation of T47D and MCF-7

T47D and MCF-7 in the logarithmic growth phase were seed on a six-well cell culture plate (1,000 cells per well), and were cultured for 24 h in the incubator with 5% CO_2_. Then, H53 and control antibody were added and cultured for 24 h, after which, the fresh 10% FBS medium were added, and the cells were cultured in an incubator at 37°C and 5% CO_2_ for 10 days. The cells were then fixed with 4% paraformaldehyde for 0.5 h after three washes, the cells were stained with crystal violet for 15 min after washing with PBS for 3 times. Clone formations were observed by using a microscope.

### The Effect of H53 on T47D and MCF-7 Cell Migration

T47D and MCF-7 cells in the logarithmic growth phase were seed into the upper chamber of the transwell at a density of 5 × 10^4^/ml. 1% FBS medium containing H53 were added into the upper chamber, the 800 µL 10% FBS was added into the lower chamber. The cells were cultured in a 37°C, 5% CO_2_ incubator for 24 h, after which, the cells in the upper chamber were removed, and the chambers were moved into 4% paraformaldehyde solution for 30 min, and then crystal violet was used to stain the cells, the microscope was used to observe and count the cells.

### Nude Mice Xenograft Experiments and Treatment Protocol

All animal care and experimental procedures were approved by the Jilin University Institutional Animal Care and Use Committee. 6–7 week old athymic nude (Nu/Nu) mice (Vital River, Beijing, China) were used in xenograft experiments. The subcutaneous xenograft tumor model established b*y* the injection of T47D (5 × 10^6^ cells/200 μL) or MCF-7 (5 × 10^6^ cells/200 μL) into the flank of mice. The athymic nude Mice were surgically implanted with estradiol pellets (0.72 mg, released over 60 days; Innovative Research of America, Sarasota, FL, United States). Implantation of the estrogen pellet was performed before mice was injected with T47D cells or MCF-7 cells. Tumors size were measured by using digital caliper, and tumor volumes were estimated by using the formula: V = [(D + d)/2]^3^, where D and d were the larger and smaller diameters, respectively. After injection of breast cancer cells, once the tumor volume reached approximately 40–55 mm^3^ the mice are randomized into groups of four–six mice per group, and the mice were treated with vehicle, IgG1 (isotype control), or H53. The tumor size was measured every 4 days using calipers. After the experiments are finished, Tumors were then harvested, fixed with 10% buffered formalin, embedded in paraffin, and subjected to pathological and immunohistochemical examinations.

### Statistical Analysis

The data are presented as mean values ± SD. The data were analyzed by One Way Variance analysis using SPSS25.0. A *p*-value of <0.05 was considered statistically significant.

## Results

### Detection of Growth Hormone Receptor/Prolactin Receptor Expression

**FIGURE 1:**
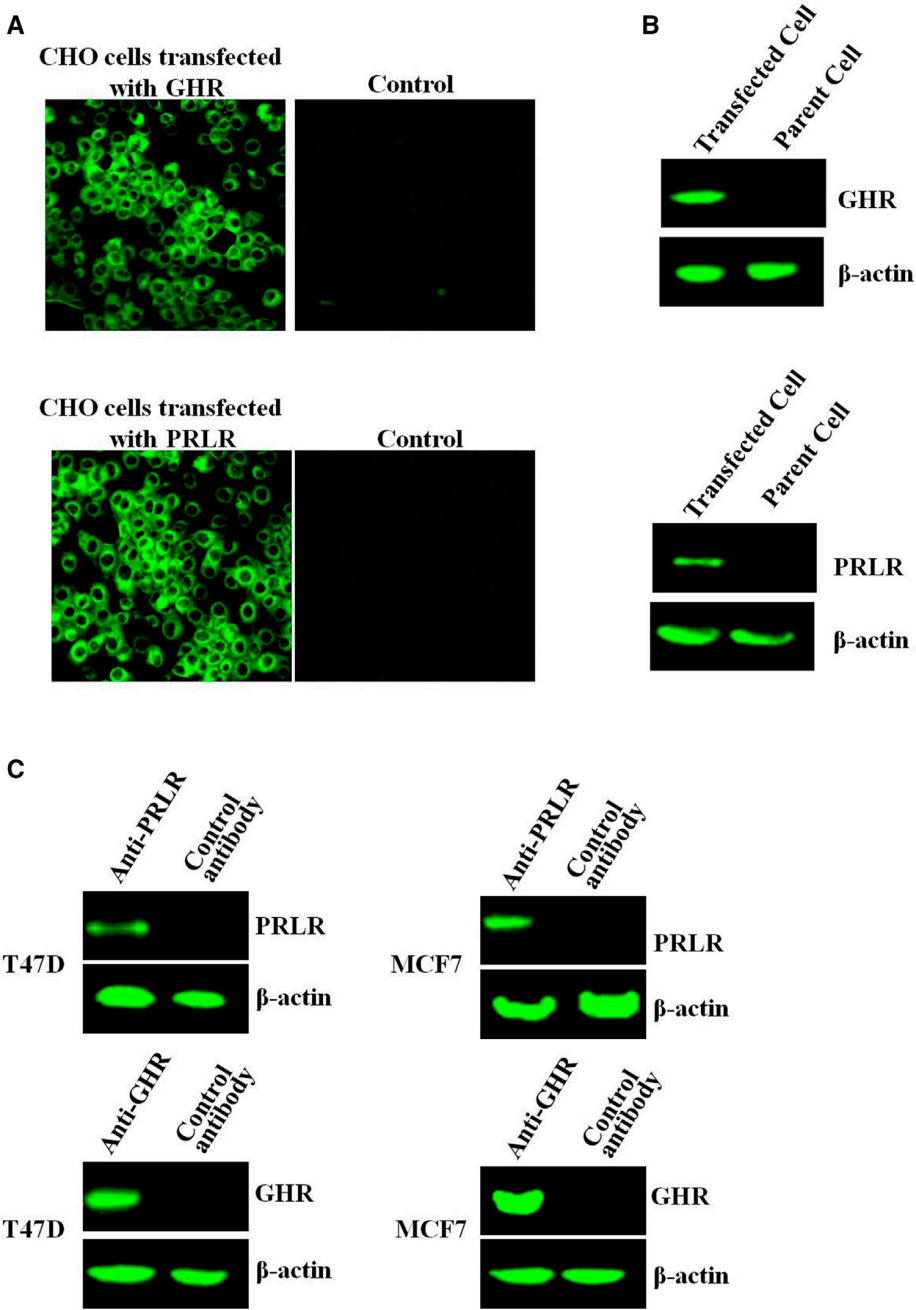
**(A)** The GHR/PRLR expression was analyzed by indirect immunofluorescence. The CHO cells transfected with GHR/PRLR were treated as described as in Materials and methods section **(B)** The GHR/PRLR expression was assessed by Western-blot. **(C)** The GHR/PRLR expression in T47D/MCF7 cells was evaluated by Western-blot analysis.

After transfection of CHO-K1 cells with PRLR or GHR, PRLR/GHR expression was evaluated by indirect immunofluorescence assay (IFA) and Western-blot, and results showed that CHO cells transfected with PRLR or GHR expressed high levels PRLR/GHR, and GHR/PRLR can not be detected in the non-transfected CHO cell (Figures 1A, B). CHO transfected with PRLR or GHR is referred to as CHO-PRLR and CHO-GHR, respectively. In addition, Western-blot analysis showed that T47D and MCF7 express both GHR and PRLR (Figure 1C).

### Screen and Characterization of Internal Image Anti-Idiotypic Antibody to GH (H53)

Human GH can bind and activate both the human GHR and PRLR. GH binds to two molecules of GHR or PRLR via its two receptor-binding regions referred to as binding sites 1 and 2, and GH binds to PRLR/GHR through its receptor-binding site 1 (Supplementary Figure S1A). In addition, the GH’s binding epitope on PRLR is overlapped with that of PRL’s receptor-binding epitope on PRLR (Goffin and Kelly, 1997). G120R is a mutated human GH molecule in which the glycine at residue 120 of hGH is mutated to arginine, resulting in that the receptor binding site 2 of GH was destroyed, therefore it has only one receptor binding site (site1) (Wang, 1996). G120R can bind to both GHR and PRLR and can antagonize signaling from both receptors. Based on the anti-idiotypic network theory of Jerne, if G120R is used as an antigen to immunize animals, an internal image anti-idiotypic antibody (Ab2β) that could mimic GH’s receptor-binding site 1 can be prepared via a series of immunological strategies. It is expected to obtain a dual-function antagonist that can simultaneously antagonize GHR and PRLR though a series of screens and identifications (Supplementary Figure S1B).

**FIGURE 2:**
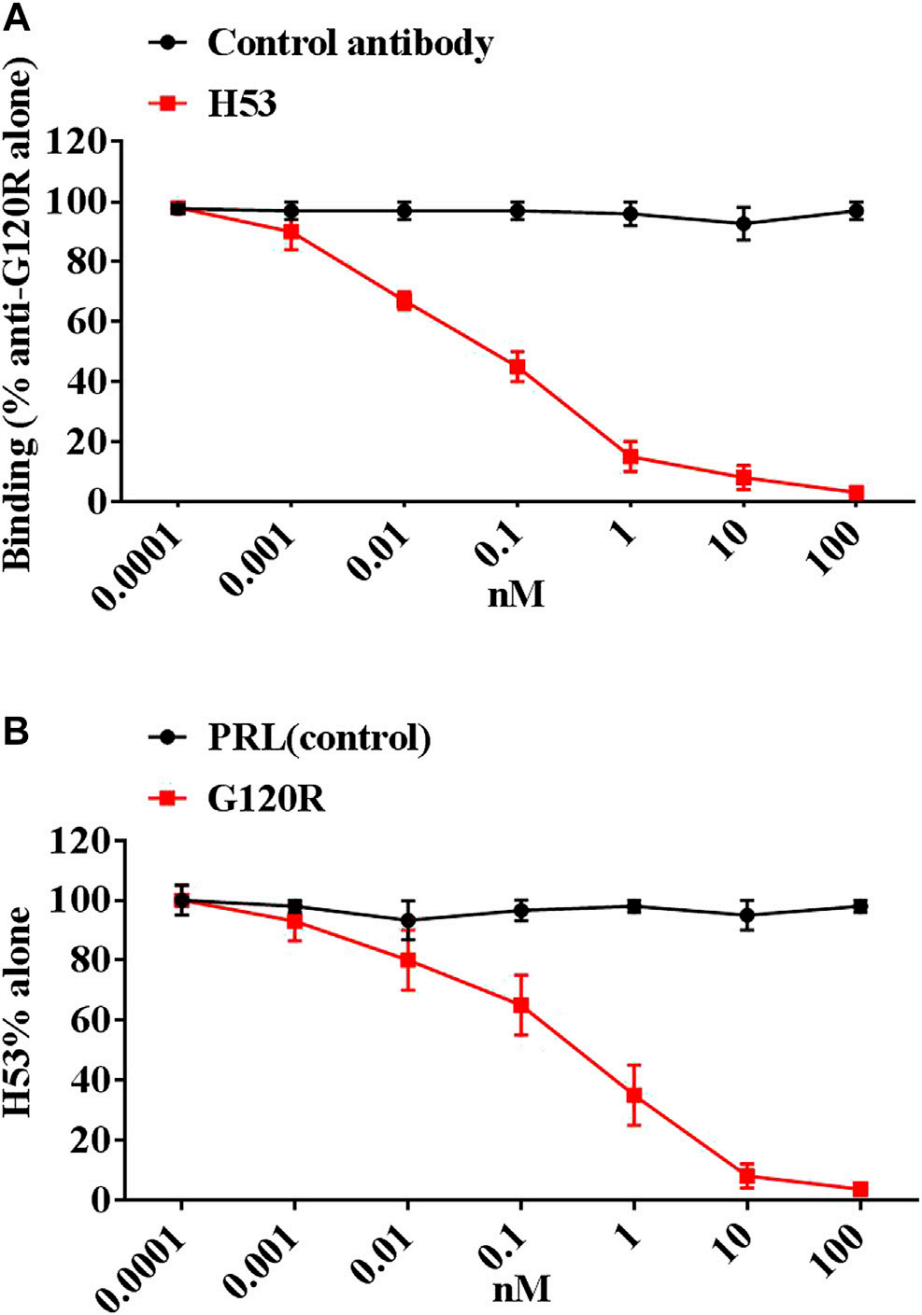
**(A)** H53 inhibited the anti-G120R by binding to GH in a dose-dependent manner. The ELISA plates were coated with G120R as described as in materials and methods section, then a constant amount of anti- G120R (namely Ab1) was mixed with different concentrations of the F(ab′)_2_ of H53. The mixtures were then added onto the ELISA plates. After three washes, IgG (Fc-specific)-HRP secondary antibody was added and incubated for 1 h, TMB was used for color development. **(B)** H53 competed with G120R to bind the anti-PRL. The microtiter plate was coated with the F(ab′)_2_ of anti-G120R antibodies, after which constant H53 and increasing concentrations of GH were added. After three washes, HRP-coupled secondary antibody was added and incubated for 1 h, color development was performed using TMB. Data are shown as the mean ± SD of three independent experiments.

After six fusions, we screened 72 potential anti-idiotype monoclonal antibodies. After further screening, we identified a potential Ab2β (named H53), which is an IgG1 antibody, H53 was purified by Protein G affinity chromatography. The purity, specificity and stability of H53 were evaluated, and the results are shown in Supplementary Material: H53 exposed at 37°C for 7 days can still bind to GHR and PRLR by flow cytometry analyses (Supplementary Figure S2); H53 exposed at 37°C for 7 days can still competitively bind to GHR or PRLR with GH or PRL (Supplementary Figure S3); H53 exposed at 37°C for 7 days can still inhibit GH/PRL-mediated signaling by Flow cytomertry (Supplementary Figure S4); H53 exposed at 37°C for 7 days could still inhibit GH-induced signaling by Western-blot analysis (Supplementary Figure S5); H53 does not bind to other growth factor receptors by ELISA assays (Supplementary Figure S6). To further characterize H53, the competitive ELISA assays were conducted to analyze if H53 exhibits typical Ab2β characteristics that could mimic an GH’s epitope, and the results showed that H53 (but not isotype-matched control antibody) inhibited anti-G120R binding to G120R in a dose-dependent manner (Figure 2A). Further experiments indicated that G120R competes with H53 for anti-G120R binding (Figure 2B). These findings indicate that H53 mimics an epitope on G120R.

**FIGURE 3:**
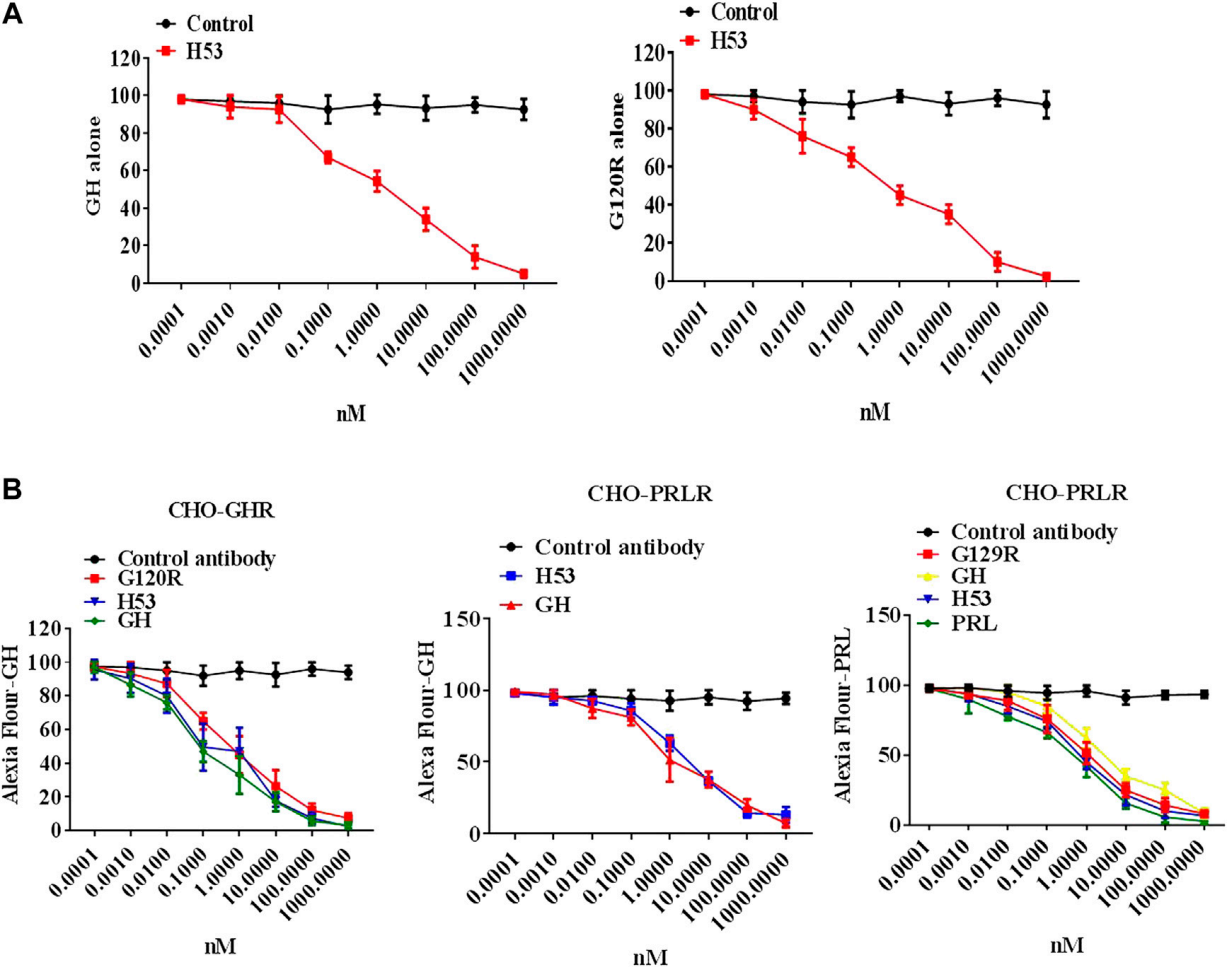
**(A)** H53 competed with GH/G120R to bind to GHR. ELISA plate was coated with the extracellular domain of GHR (GHR-ECD), and then GH alone or GH was mixed with increasing concentrations of F(ab')_2_ of H53 or F(ab′)_2_ of control antibody. Next, the mixtures were added to each well of the ELISA plate. **(B)** H53 inhibited GH/PRL binding to GHR/PRLR as determined by the competitive receptor binding assay. The experimental process has been described in detail in the materials and methods section. The cell samples were examined using a Becton-Dickinson FACS Calibur Flow Cytometer; Data are shown as the mean ± SD of three independent experiments.

To evaluate whether H53’s receptor-binding epitope overlaps with that of G120R, we carried out a competitive ELISA assay, and the results showed that H53 competes with GH/G120R for GHBP binding in a dose-dependent manner (Figure 3A) (GHBP belongs to the extracellular domain of GHR, so GHBP can still interact with GH). Furthermore, a competitive receptor binding assay was done to test whether H53 competitively binds GHR/PRLR with GH/PRL. As shown in Figure 3B, in CHO-GHR cell model, it can be seen that non-fluorescently labeled GH, G120R and H53 (but not control antibody) can inhibit Alexa-flour-GH binding to GHR in CHO-GHR cells. In addition, GH and H53 (but not control antibody) could inhibit Alexa-flour-GH binding to PRLR in CHO-PRLR cells. Furthermore, in CHO-PRLR cell model, PRL, G129R (des 1–9), GH and H53 (but not control antibody) can inhibit Alexa-flour-PRL binding to PRLR. Taken together, these results indicated that H53’s receptor-binding epitope is overlapped with the GH/PRL’s receptor-binding site.

**FIGURE 4:**
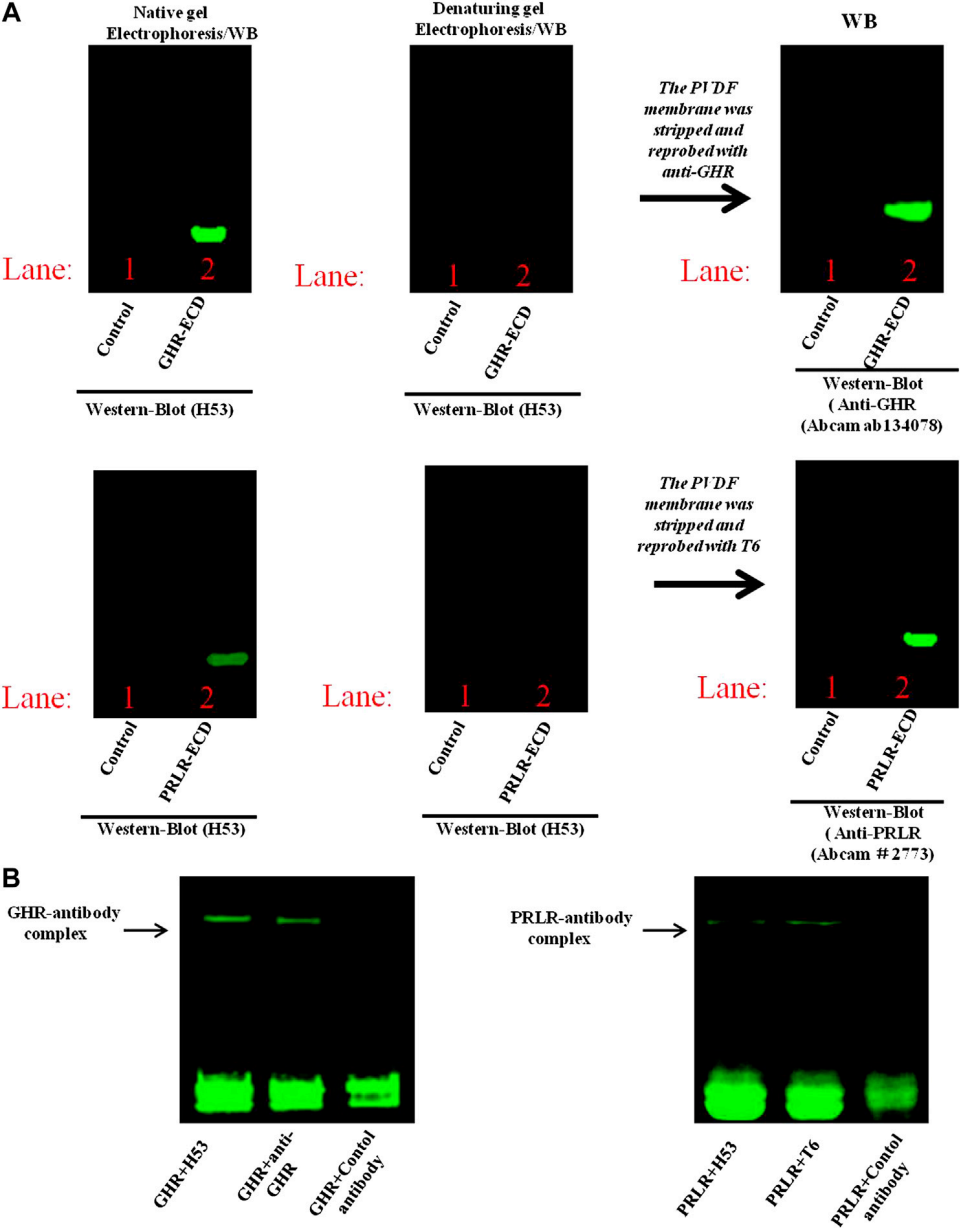
**(A)** Analysis of epitope recognized by H53 on GHR-ECD or PRLR-ECD. Denatured and non-denatured PRLR-ECD or GHR-ECD were subjected to SDS-PAGE and transferred to PVDF membrane. After washing and blocking, the membranes were incubated with H53, and incubated with Alexa-488-labeled goat anti-mouse for 1 h. After washing, the membranes were analyzed using the Bio-Rad fluorescence detection system. **(B)** Detection of direct interaction between H53 and PRLR-ECD/GHR-ECD by native gel electrophoresis. BIO-Rad ChemiDoc XRS Gel Imaging System was used to detect the signal.

To further characterize the binding epitope of H53 on PRLR/GHR, the GHR-ECD/PRLR-ECD was subjected to denaturing and non-denaturing electrophoresis and analyzed by western blot analyses, the results showed that H53 recognizes non-denatured GHR-ECD/PRLR-ECD, but not the denatured GHR-ECD/PRLR-ECD (Figure 4A), indicating that H53 recognizes the conformational epitope of GHR/PRLR, which is similar to GH (studies have shown that GH recognizes the conformational epitope on GHR/PRLR (Chilton and Hewetson, 2005). Moreover, analysis of the interaction between H53 and natural GHR/PRLR using native gel electrophoresis revealed that H53 could interact with non-denaturing GHR-ECD/PRLR-ECD (Figure 4B). In addition, to further determine whether H53 directly and specifically binds to GHR and PRLR, we conducted IP-WB, and results demonstrated that H53 binds to GHR/PRLR (Supplementary Figure S7).

Affinity analysis by SPR revealed that the affinity constant of H53 was 1.5 × 10^−7^ M (for GHR) and 1.2 × 10^−6^ M (for PRLR), and the affinity of GH was 1.85 × 10^−7^ M (for GHR) and 1.53 × 10^−6^ M (for PRLR), which is similar to that measured with H53.

### H53’s Cell Behavior

**FIGURE 5:**
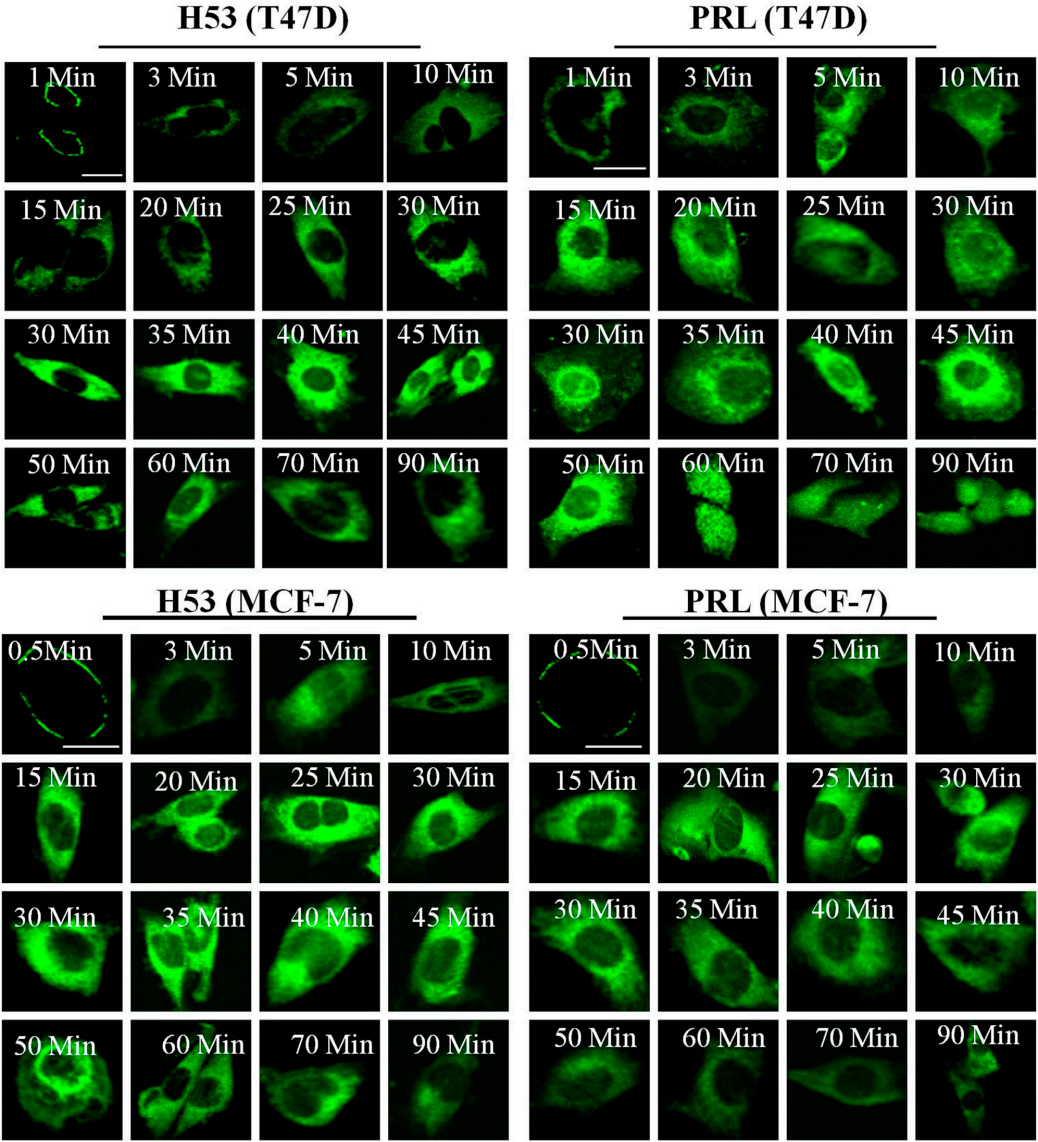
Internalization and trafficking of H53. The cells were serum-starved for 6 h, and then the cells were incubated with fluorescent labeled-PRL, GH or H53 for different time points. After washing, the cell samples were detected by using a confocal laser scanning microscopy (Olympus FV3000).

We explored the cellular behavior of H53 on T47D and MCF-7 cells, and results showed that H53 is internalized into cells in a time-dependent manner. At 0–1 min, H53 mainly localizes on the cell membrane. As time passes, cytoplasmic fluorescence gradually increases, and at 30–45 min, fluorescence reaches a maximum intensity (Figure 5, left panel). We also analyzed PRL/GH’s internalization and observed that PRL/GH uptake into cells increases over time, reaching maximum intensity at 30–50 min(Figure 5, right panel). Next, we preliminarily investigated the intracellular sorting mechanism of H53 and found that H53 entered into the EEA1-positive endosome (Supplementary Figure S8). Further studies revealed that H53 also enters into Rab5/7 and LAMP1-postive endosome (Supplementary Figure S9). Taken together, these observations suggest that H53 may share the same endocytosis and intracellular sorting mechanism as PRL.

### H53 Inhibits Growth Hormone Receptor/Prolactin Receptor-Mediated Signaling in CHO-Prolactin Receptor and CHO-Growth Hormone Receptor Cells

**FIGURE 6:**
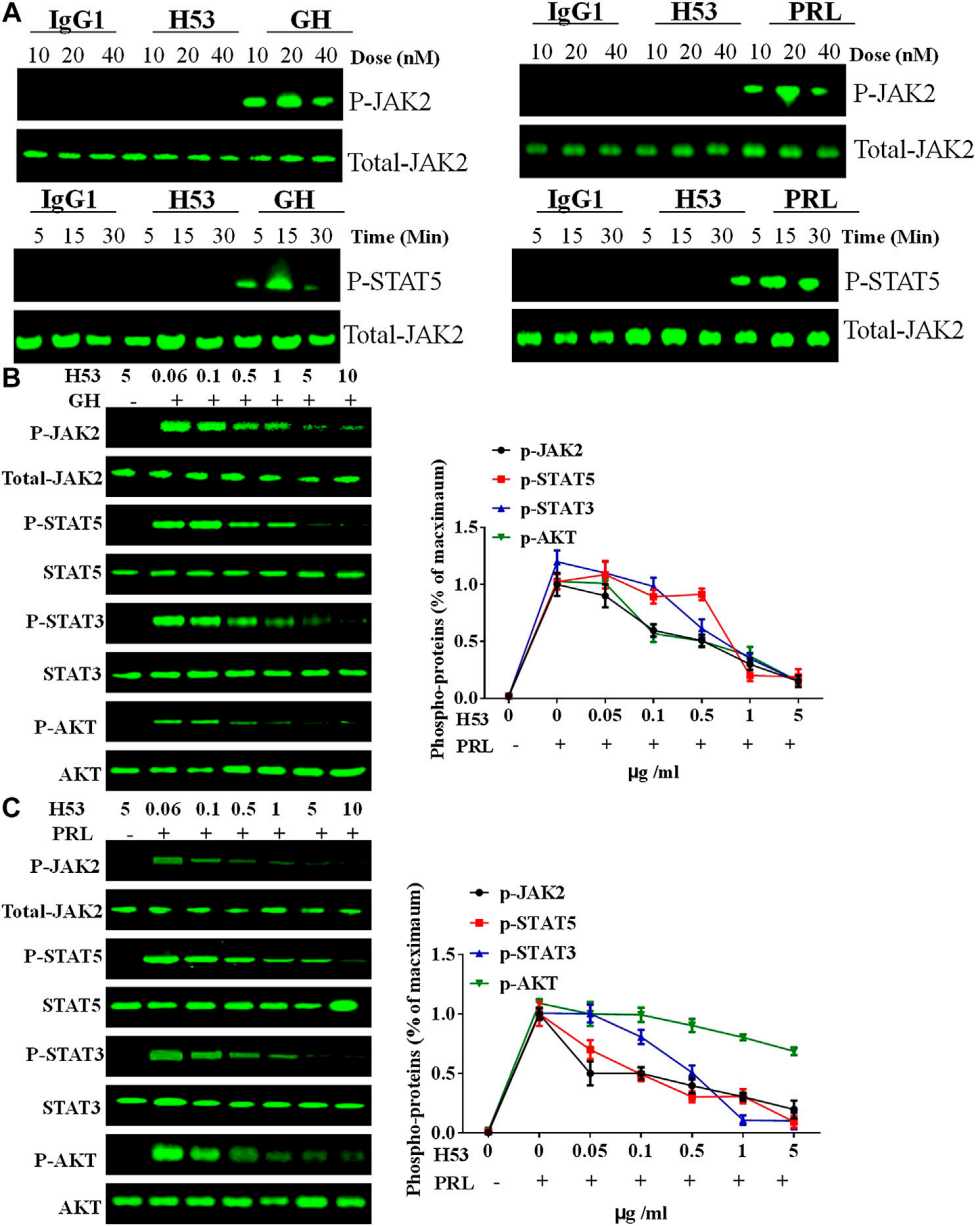
**(A)** H53 has no agonistic activities. CHO-PRLR or CHO-GHR was starved for 6 h. The cells were washed with sterile PBS, and then stimulated with different concentrations of H53, IgG1 (isotype control) or PRL/GH (positive control). Western-blot analysis was then performed to measure proteins expression. **(B)** Inhibition of GH-induced signaling pathway by H53 but not control antibody. **(C)** Inhibition of PRL-induced signaling pathway by H53 but not control antibody. Results shown are representative of at least three independent experiments. ImageJ software was used to quantify the optical density of protein bands. Data are presented as the mean ± SD of three independent experiments. Cells challenged with PRL/GH alone were considered as 100%.

The above experiments show that H53 specifically binds to GHR and PRLR. We further analyzed whether H53 possesses the properties as GHR or PRLR antagonist. GH/GHR or PRL/PRLR initiates its biological functions by activation of intracellular signaling molecules (Chilton and Hewetson, 2005). First, we ruled out that H53 can activate intracellular signaling pathways mediated by GHR or PRLR. The effect of H53 on GHR/PRLR-mediated intracellular signaling were evaluated, and the results showed that at various concentrations and different time points, H53 and isotype control antibody did not activate GHR/PRLR-mediated intracellular signaling (JAK2-STAT5 signaling) (Figure 6A). In contrast, GH/PRL activates GHR or PRLR-mediated signaling pathway.

Next, we analyzed the effect of H53 on GH or PRL-induced intracellular signaling. GH or PRL alone strongly activates STAT5, STAT3 and AKT signaling in CHO-GHR or CHO-PRLR cells. However, when cells were treated with various concentrations of the premixes of H53/GH or H53/PRL, GHR/PRLR-mediated signaling were significantly inhibited, and the inhibitory activity of H53 is increased with increasing concentrations (Figures 6B, C). In addition, the control antibody could not inhibit GHR/PRLR-mediated signaling (Supplementary Figure S10). Furthermore, EMSA assays indicated that H53 inhibits STAT5’s DNA binding capacity (Supplementary Figure S11). Furthermore, IFA results found that H53 inhibits STAT5 nuclear transport induced by PRL/GH (Supplementary Figure S12). Taken together, these findings show that H53 inhibits GH/PRL -mediated signaling.

### H53 Exhibits Antagonistic Activity in Tumor Cell Models

**FIGURE 7:**
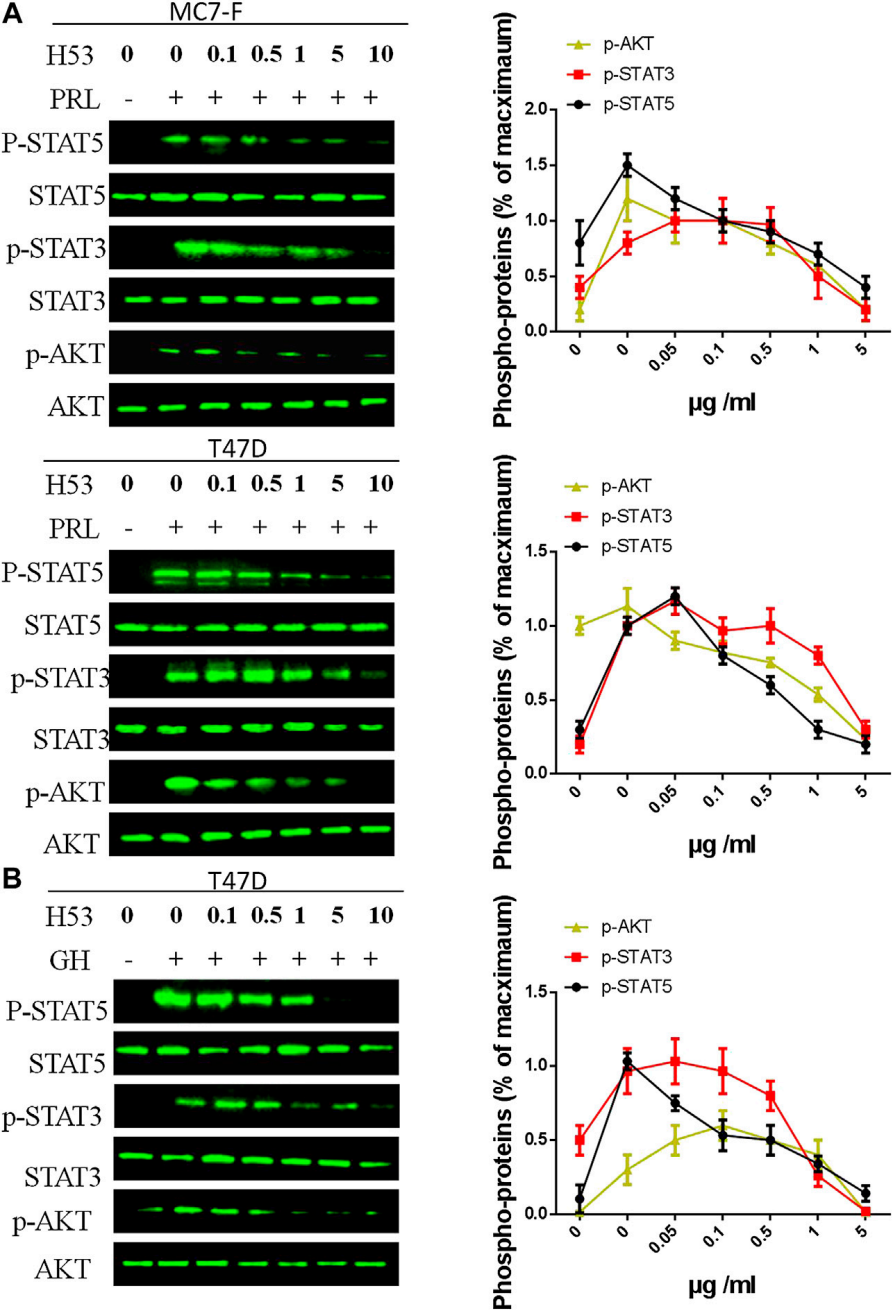
**(A,B)** Inhibition of PRL-induced signaling pathway by H53. MCF-7 and T47D cells were starved for 12 h. Cells were washed with sterile PBS and treated with PRL alone or mixture containing different concentrations of H53 and constant PRL for the indicated durations. Next, cells were washed and used for western-blot. **(C)** Inhibition of GH-induced signaling pathway by H53. ImageJ software was used to quantify the optical density of protein bands. Data are presented as the mean ± SD of three independent experiments. The cells were challenged with PRL alone were considered 100%.

As shown in Figure 7A, H53 showed a dose-dependent inhibition of PRLR-mediated signaling induced by PRL in MCF7 and T47D cells. H53 inhibited PRL-mediated signaling in a dose-dependent manner. Our analysis indicated that at a concentration of 0.5 μg/ml H53, PRLR-mediated signaling was suppressed, and inhibitory effect reached nearly 50% at 5 μg/ml H53 concentration. At a H53 dosage 5–10 g/ml, PRLR-mediated signaling was almost 100% inhibited. Furthermore, H53 also inhibited GHR-induced intracellular signaling pathway (Figure 7B). In addition, the control antibody could not inhibit GHR/PRLR-mediated signaling (Supplementary Figure S13). Additionally, EMSA assays indicate that H53 inhibits STAT5’s DNA binding capacity (Supplementary Figure S14). IFA results found that H53 inhibits STAT5 nuclear transport induced by PRL (Supplementary Figure S15).

### Cell Proliferation Induced by PRL Is Inhibited by H53

**FIGURE 8:**
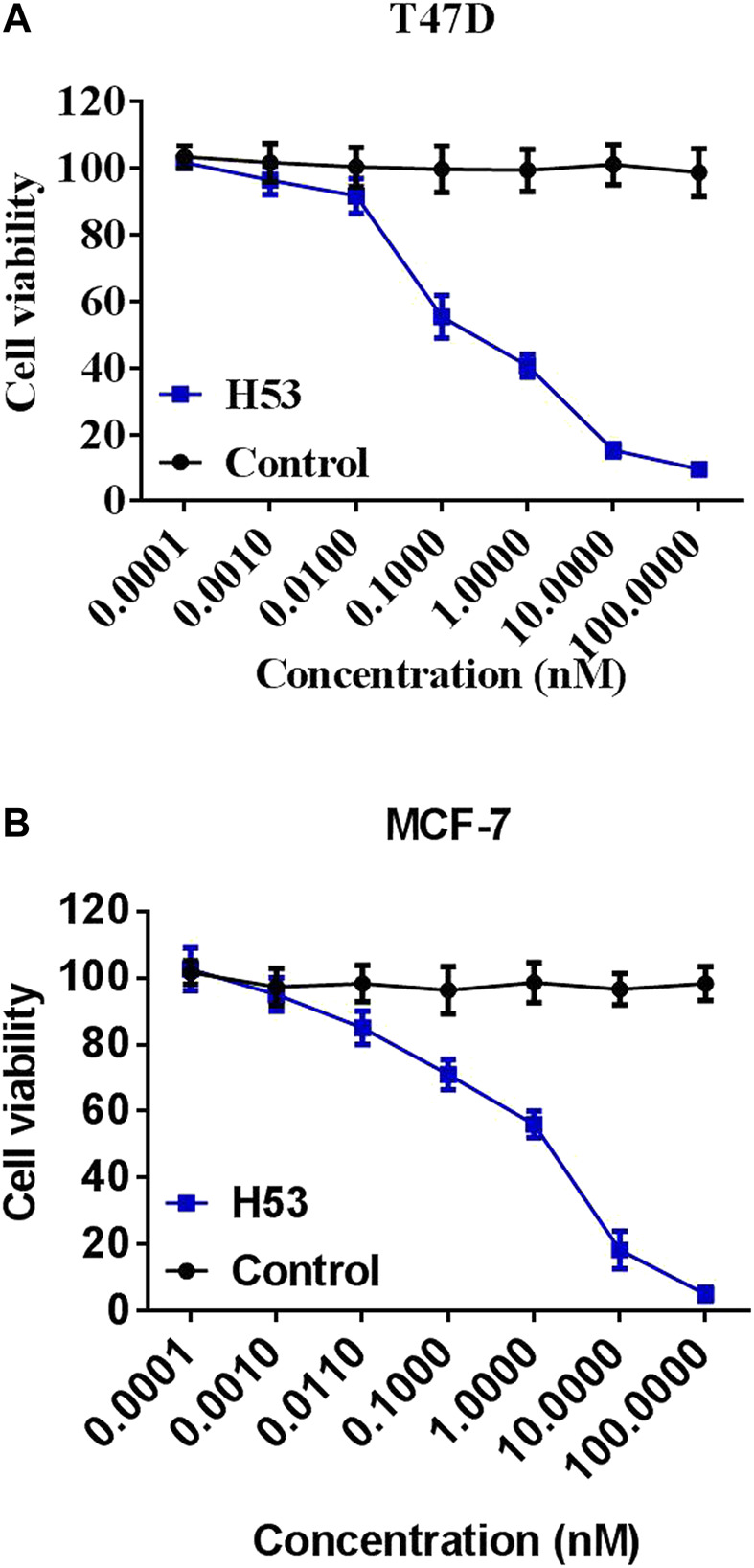
Inhibition of the cell proliferation. MCF-7 **(A)** and T47D **(B)** cells were dispersed in a 96-well culture plate (100ml/well) at a cell density of 1 × 10^4^cells/well. A mixture of constant H53 and an increasing concentrations of H53 or an isotype-matched control antibody were added to the cells on culture plate. The plate was incubated at 37°C, 5% CO_2_ for 24 h. These cells were used to perform the CCK-8 assay. The absorbance of the plate was read using a microplate reader at 450 nm. Date are presented as the mean ± SD of three independent experiments.

To further characterize the antagonistic activity of H53, we carried out cell proliferation assays, and the results showed that H53 significantly inhibits the proliferation of MCF7 and T47D cells induced by PRL in a dose-dependent manner. The control antibodies did not exhibit antagonistic activity (Figure 8). 

**FIGURE 9:**
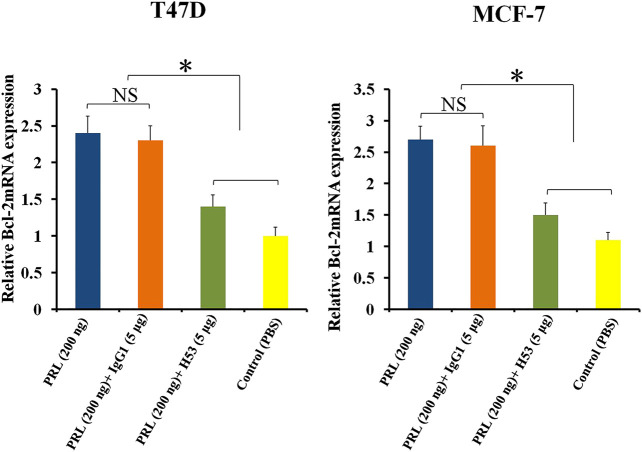
H53 suppressed the expression of Bcl-2 gene. Bcl-2 mRNA level was measured with RT-qPCR assay. T47D and MCF-7 were challenged with PRL (200 ng/ml), the mixture of PRL/H53 or the mixture of PRL/IgG1. Asterisk (*) represents a statistically significant (*p* < 0.05).

Bcl-2 is an anti-apoptotic gene (Beck et al., 2002). Previous studies have shown that PRL up-regulates BCL-2 expression in tumor cells (Beck et al., 2002). In this study, we found that H53 suppresses BCL-2 expression (Figure 9).

### H53 Inhibits the Cloning Formation of T47D and MCF-7

**FIGURE 10:**
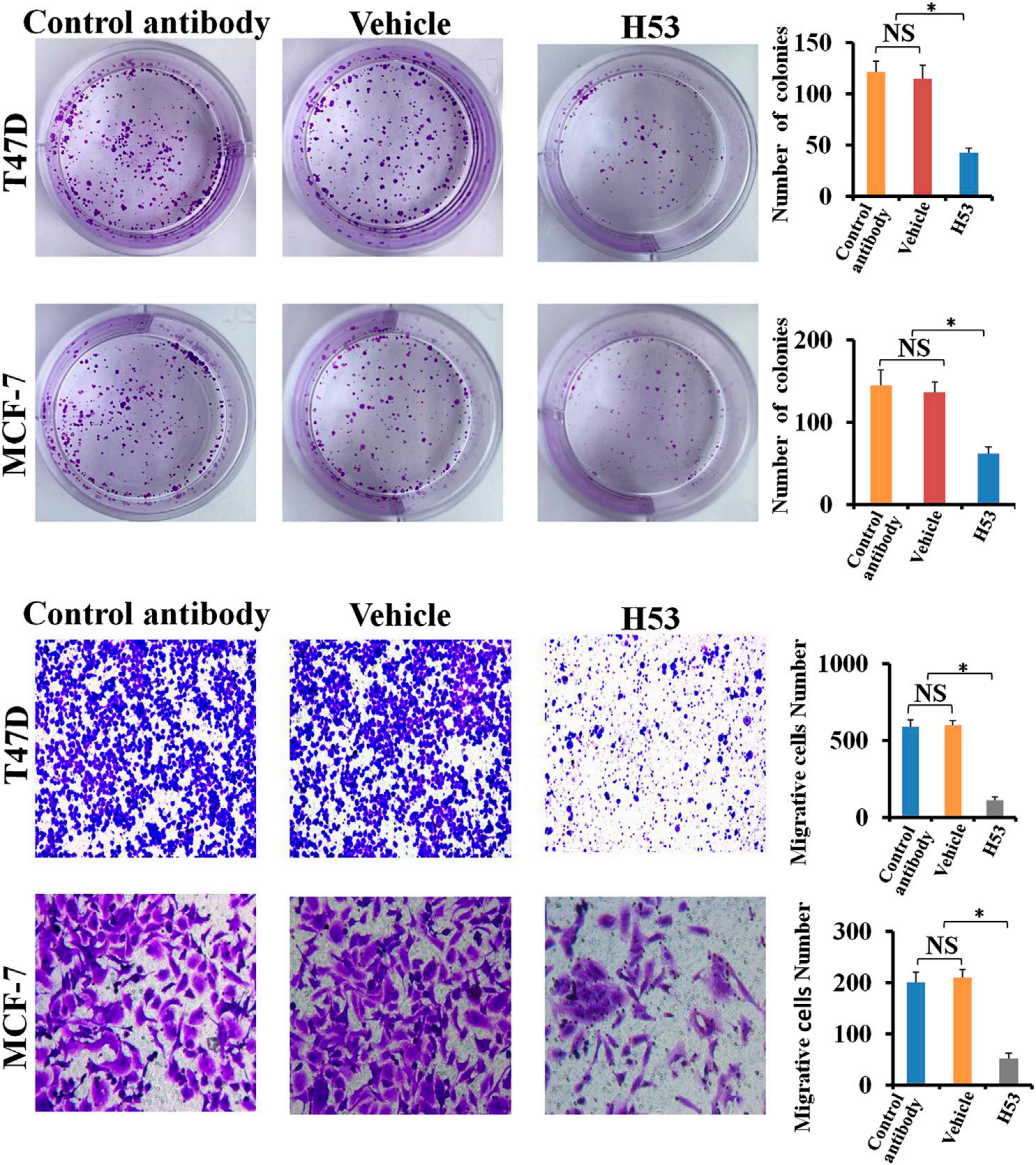
**A)** H53 inhibits the cloning formation ability of T47D and MCF-7. The experimental process has been described in detail in the materials and methods section. **(B)** Transwell assay was performed to determine the effect of H53 on the migration abilities of T47D and MCF-7 cells. Asterisk (*) represents a statistically significant (*p* < 0.05).

Clone formation was performed to further detect the antagonistic activity of H53, and the results showed that the cloning formation ability of H53-treated cells was significantly inhibited (Figure 10A). Next, we further investigated the effect of H53 on cell migration of MCF-7 and T47D. It can be seen that H53 (but not isotype control antibody) inhibited cell migration of MCF-7 and T47D (Figure 10B).

### H53 Induces Growth Hormone Receptor/Prolactin Receptor Down-Regulation

**FIGURE 11:**
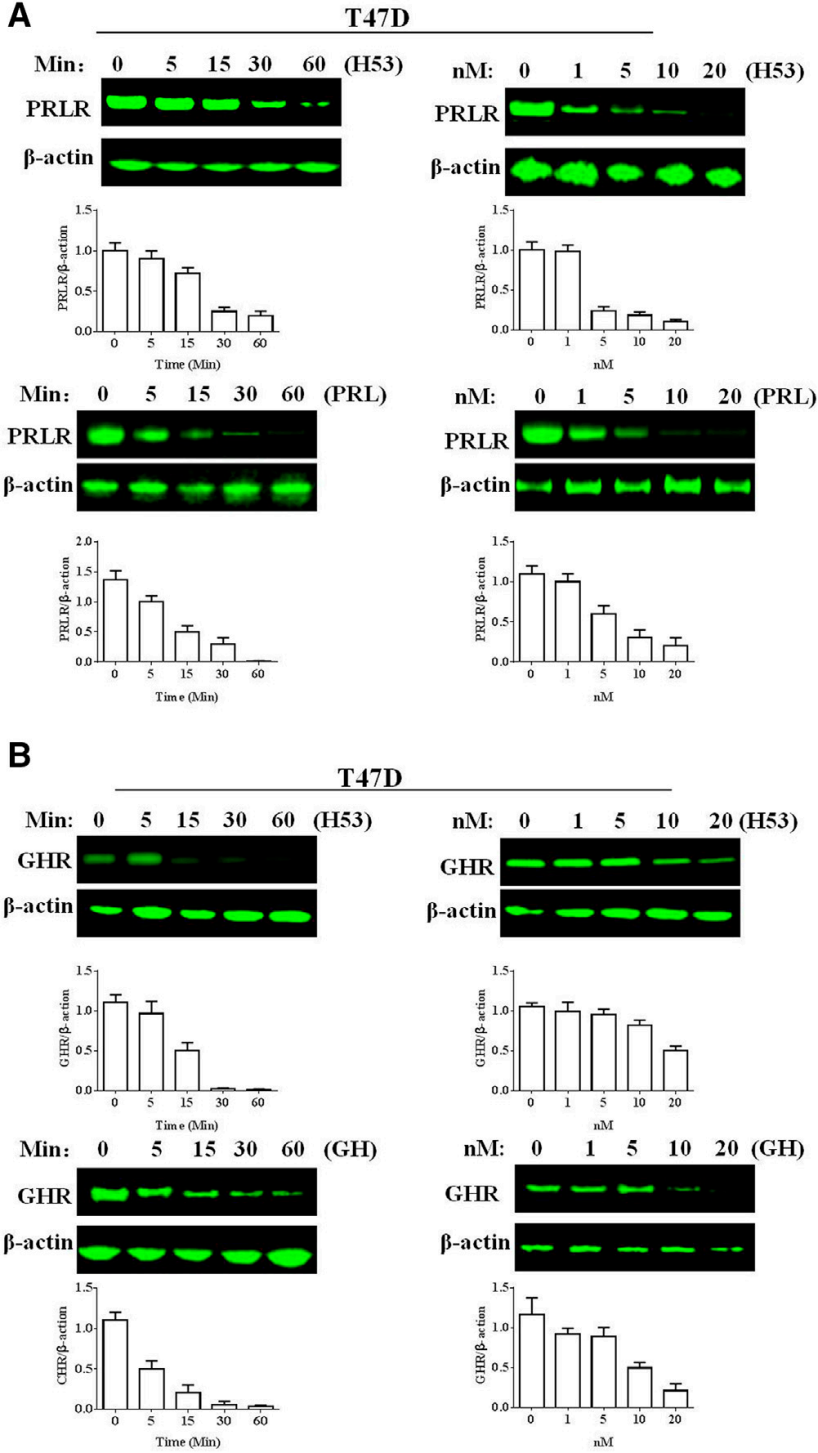
**A)** H53 down-regulated PRLR/GHR expression in T47D cells. The cells were treated with H53 at the indicated dose and durations. Proteins were isolated from the treated cells for Western blotting. **(B)** H53 induced GHR down-regulation in T47D cell. Data are presented as the mean ± SD of three independent experiments.

Next, we analyzed if H53 downregulates PRLR/GHR in T47D cells, and the results revealed that H53 induces PRLR down-regulation in a time and dose-dependent manner (Figure 11A). In addition, H53 also induced GHR down-regulation in T47D cell (Figure 11B).

### Inhibition of the Growth of T47D and MCF-7 Xenografts by H53 *in vivo*

To explore the *in vivo* effect of H53, the subcutaneous xenograft tumor model was established by the injection of T47D (5 × 10^6^ cells/200 μL) or MCF-7 (5 × 10^6^ cells/200 μL) into the flank of mice. When the tumor volume reached approximately 40–55 mm^3^, the mice are randomized into groups of four–six mice per group, and the mice were treated with vehicle, IgG1 (isotype control antiboy), or H53. The results showed that H53 significantly inhibited the growth of T47D and MCF-7 xenografts, but control antibody (IgG1) has no effect. Furthermore, immunohistochemical staining also showed that p-STAT5/p-STAT3/p-AKT level were also down-regulated in H53-treated xenograft tumor compared to vehicle or IgG1-treated xenograft tumor. Furthermore, immunohistochemical staining results indicated that the cell proliferation marker (Ki67) was down-regulated in H53-treated xenograft tumor compared to vehicle or IgG1-treated xenograft tumor. In addition, TUNEL assay showed that apoptosis was increased in H53-treated xenograft tumor when compared to IgG1-treamted xenograft tumor.

## Discussion

In 1974, an immunologist Jene proposed immune network theory (Jerne et al., 1992; Clevenger et al., 2008; Xu et al., 2013), which states that antigens stimulate the body to produce corresponding antibodies (called Ab1), and the variable region of Ab1 itself can be used as an antigen which induces the production of anti-antibodies against Ab1. These antibodies are called anti-idiotypic antibodies (Ab2). Anti-Id is divided into four types: Ab2α, Ab2γ, Ab2ε, and Ab2β. The structural characteristics of Ab2β mimick the original antigen, and is thus referred to as “internal image of initial antigen.” In this study, we prepared a anti-idiotypic antibody (Ab2β), termed as H53, which shows the potential of GHR/PRLR dual-function antagonists, and H53 exhibited the anti-tumor effects.

In general, Ab2β is considered as the “internal image” of hormones or cytokines. For this reason, anti-idiotypic antibody strategies are generally used to prepare antigen mimics (Chen et al., 1999). In previous studies, Amit et al. (1986) prepared a polyclonal anti-idiotypic antibody to PRL which could mimick PRL’s receptor-binding sites (Beck et al., 2002; Meng et al., 2016), and thus it can interact with PRLR. In addition, Lan et al. reported that anti-idiotypic antibodies of GH can activate GHR and elicit similar biological activities as GH (Hainan et al., 2015a; Hainan et al., 2015b). Furthermore, they observed that a series of prepared anti-idiotypic antibodies to GH exhibited antagonistic activity (Hainan et al., 2015b). Based on this, they proposed that anti-idiotypic antibody can also serve as antagonists. Thus, anti-idiotypic antibody strategy can be used as an alternative strategy for the preparation of GHR/PRLR inhibitor. Traditionally, GH/PRL is converted from an agonist to an inhibitor mainly by introduction of different mutations in its polypeptide chain (Wang, 1996). Thus, the mutated GH/PRL analog can competitively inhibit endogenous GH/PRL by binding to GHR/PRLR. Among GHR/PRLR antagonists, including G120R/G120K-hGH/B2036 (for GHR) and G129R-hPRL/S179D-hPRL/Δ1-9-G129R-hPRL (for PRLR) are widely studied (Goffin and Kelly, 2005). The first report on PRL analog antagonists was released in the early 1990s. Goffin et al. replaced the glycine (G) in hPRL helix 3 with arginine (R) to design PRL receptor antagonists (Goffin and Kelly, 2005), the development of G129R is mainly based on the development strategy of GHR antagonist G120R (Clevenger et al., 2008). First-generation GHR/PRLR antagonist are termed as G120R-hGH and G129R-hPRL, respectively. Further research showed that G129R-hPRL can inhibit tumor cell proliferation (Chen et al., 1999). In addition, G129R-hPRL promotes the secretion of TGF-β1 (an apoptosis factor) (Chen et al., 1999). It was later recognized that G129R-hPRL can activate caspase-3 inducing apoptosis and down-regulating expression of anti-apoptotic gene bcl-2 (Beck et al., 2002). Similarly, G120R has experienced a similar research process (Langenheim et al., 2006; Clevenger et al., 2008). In subsequent studies, researchers found that G129R-hPRL or G120R-hGH has a weak activity, thus its antagonistic activity is still controversial (Wang, 1996). Since then, researchers have attempted to develop “pure” PRLR antagonists, such as Δ1-9-G129R-hPRL (Goffin and Touraine, 2015). So far, PRLR antibody has been identified as another class of antagonists. To date, however, there is no commercially available PRLR antagonist. [The commercial antagonist of growth hormone receptor is pegvisomant (Hainan et al., 2015b)].

**FIGURE 12:**
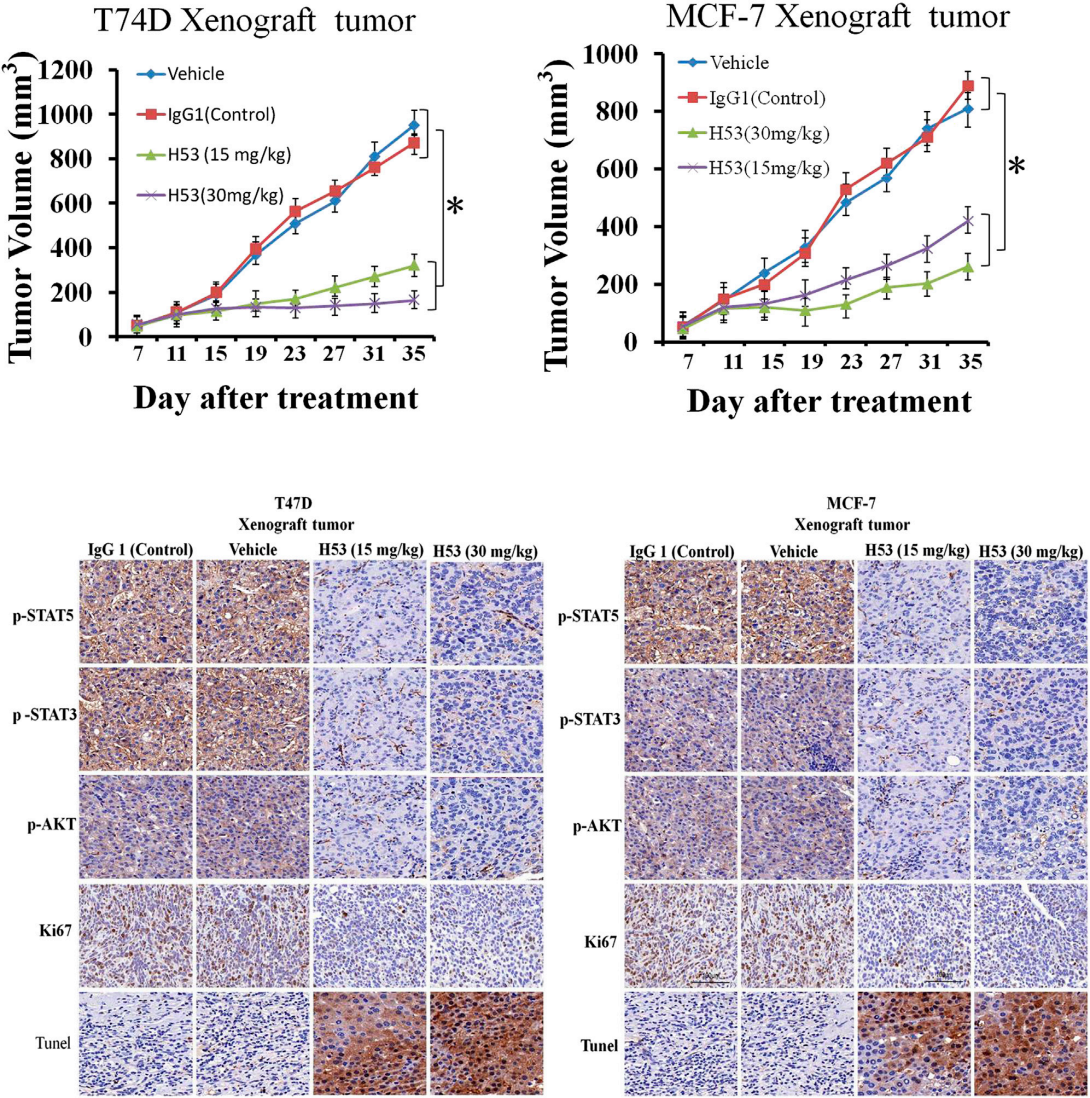
**(A)** H53 (but not control antibody) inhibited the growth of T47D and MCF-7 xenografts. The xenograft experiments were performed as described in the Material and methods section. Mice were treated with vehicle, IgG1 (isotype control), or H53 twice per week. Data represent the mean ± SD. *p < 0.05 vs. vehicle or IgG1 control. **(B)** Immunohistochemical staining for Ki67 of tumor sections from T47D and MCF-7 xenografts after treatment with 30 mg/kg IgG1 or 30 mg/kg H53.

Studies have shown that the occurrence and development of breast cancer are closely related to GH/PRL, and PRLR and GHR are co-expressed on breast cancer. Furthermore, recent study from Frank’s team shows that there is a GHR-PRLR hybrid receptor on breast cancer. Therefore, it is required to develop a dual-acting inhibitor of GHR/PRLR. Although previous studies have found that G120R can be used as a dual-effect inhibitor against PRLR/GHR, a series of studies have shown that G120R itself may make a weak activator (Goffin and Kelly, 2005). In the current study, we found that H53 showed good antagonistic properties *in vivo* and *in vitro*, not only inhibiting GHR/PRLR-mediated intracellular signals, but also blocking breast tumor proliferation (Figures 6–12). H53 alone did not exhibit any agonistic characteristics. The current study suggests that H53 may be a potential GHR/PRLR bispecific inhibitor.

In conclusion, ccurrent research shows that H53 is a new inhibitor of GHR/PRLR which has potential as a reagent for the treatment of breast tumors. Evidence also shows that anti-idiotypic antibody strategy is a feasible approach for preparing antagonists related to growth factors or hormones.

## Data Availability Statement

The original contributions presented in the study are included in the article/Supplementary Material, further inquiries can be directed to the corresponding author/s.

## Author Contributions

Conceived and designed the experiments: JW. Performed the experiments: XC, DW. Analyzed the data: YZ, XL

## Funding

This work was supported by the National Natural Science Foundation (Grant number 81601045).

## Conflict of Interest

The authors declare that the research was conducted in the absence of any commercial or financial relationships that could be construed as a potential conflict of interest.
