# Supplementary figures and images for: Preparation of a Growth Hormone Receptor/Prolactin Receptor Bispecific Antibody Antagonist Which Exhibited Anti-Cancer Activity
Source: Front Pharmacol. 2020 Dec 10;11:598423. doi: 10.3389/fphar.2020.598423 (PMC7759028; doi:10.3389/fphar.2020.598423)

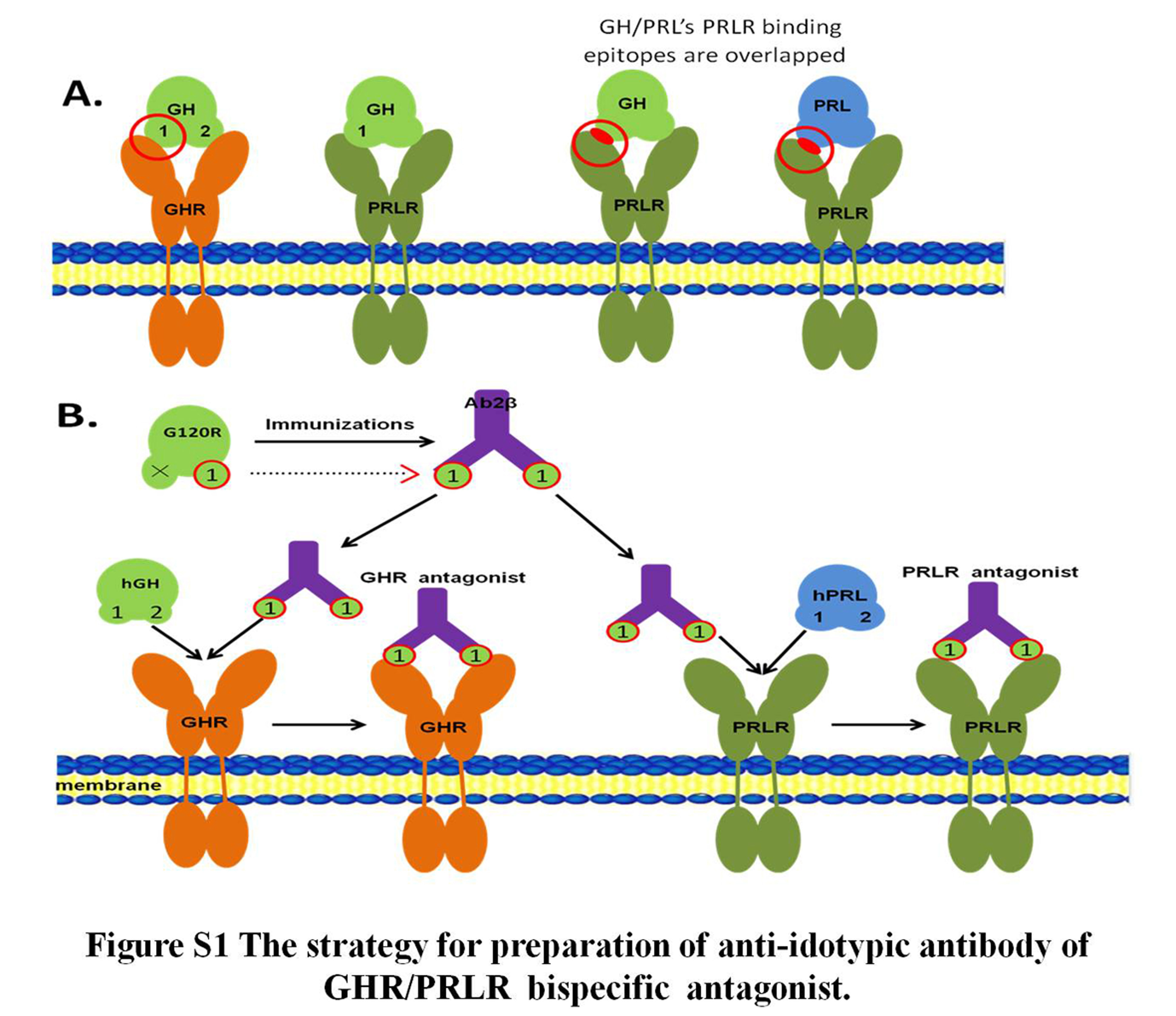

Supplement: Supplementary file 1 [file image1.jpeg]

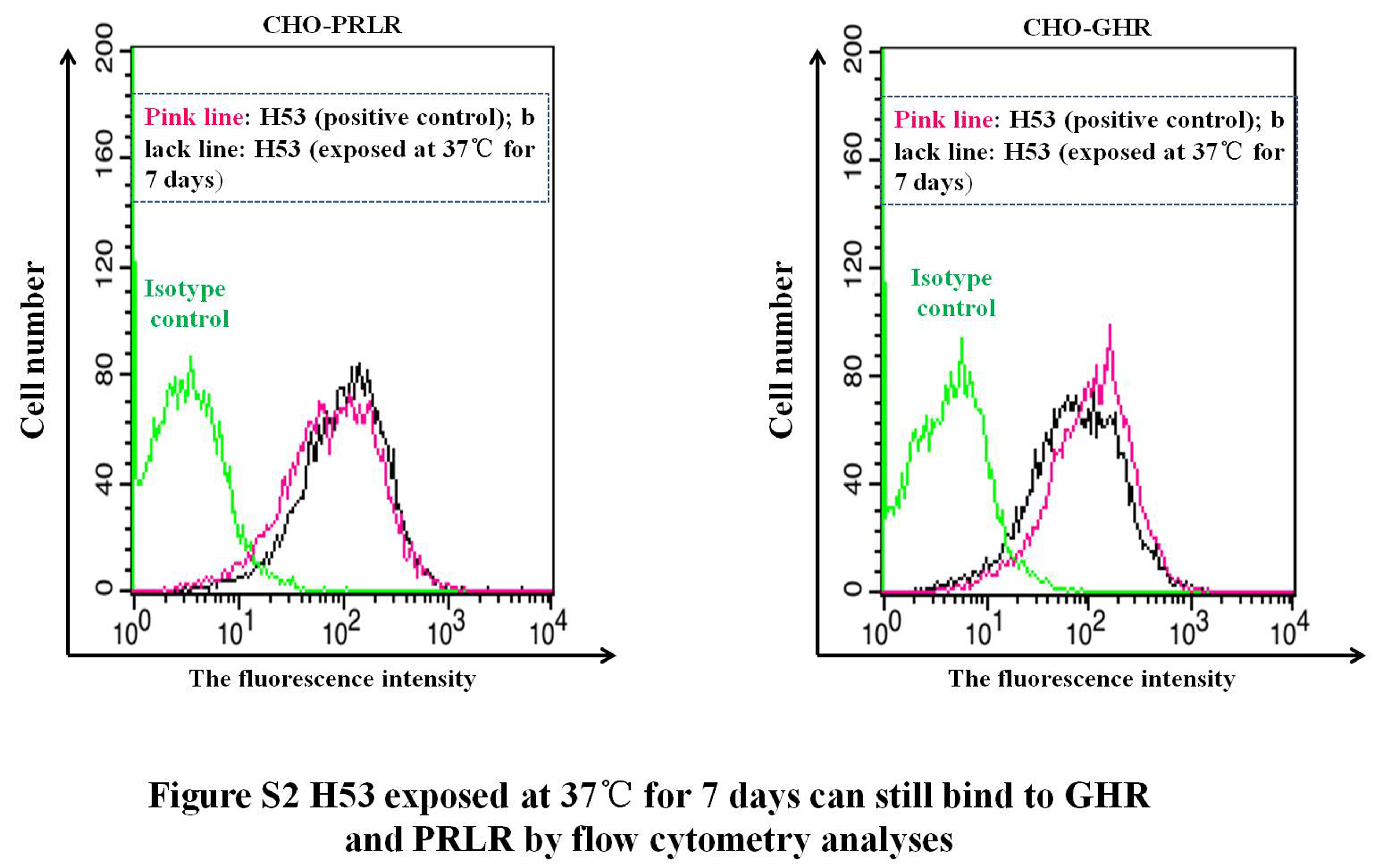

Supplement: Supplementary file 2 [file image2.jpeg]

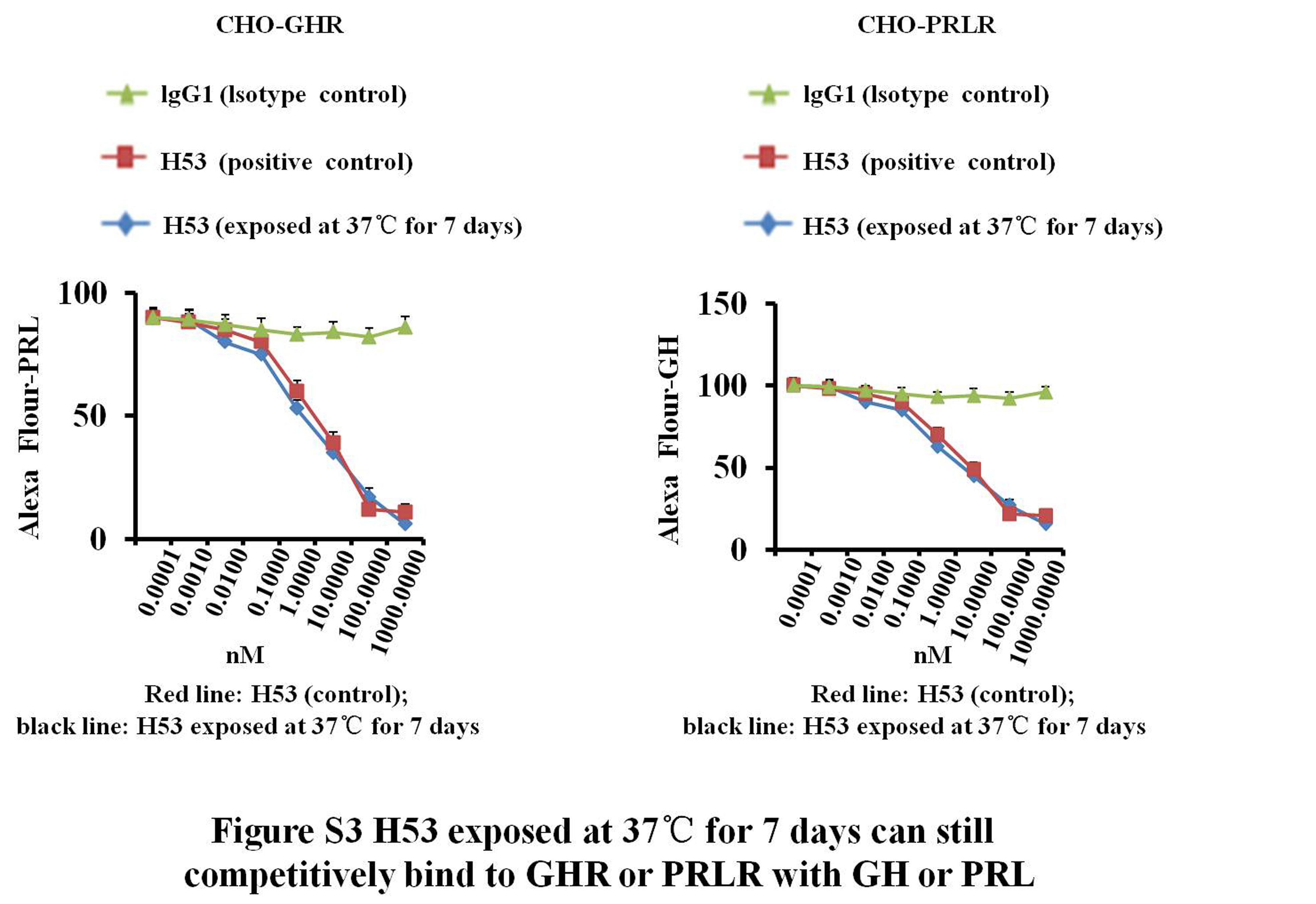

Supplement: Supplementary file 3 [file image3.jpeg]

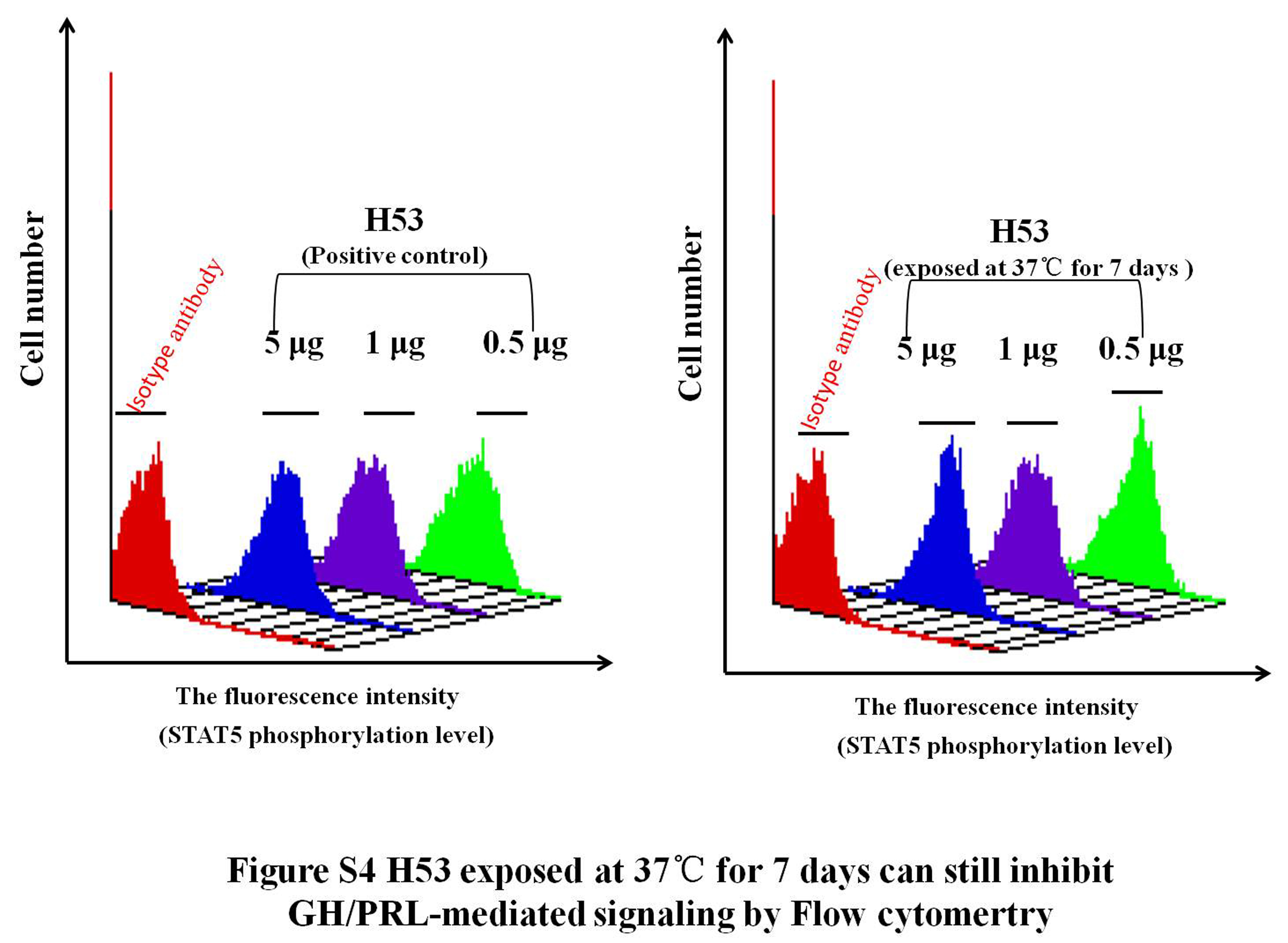

Supplement: Supplementary file 4 [file image4.jpeg]

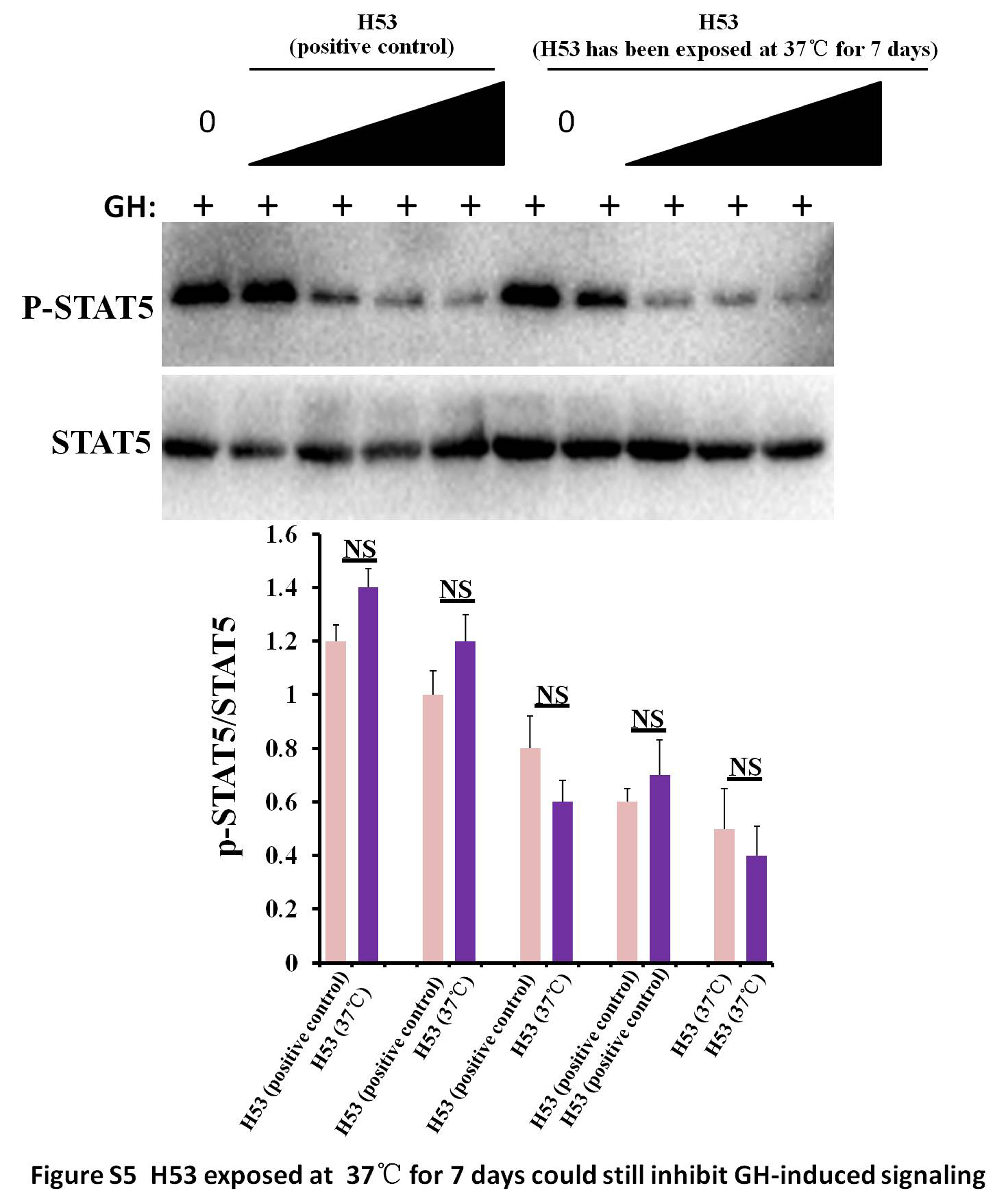

Supplement: Supplementary file 5 [file image5.jpeg]

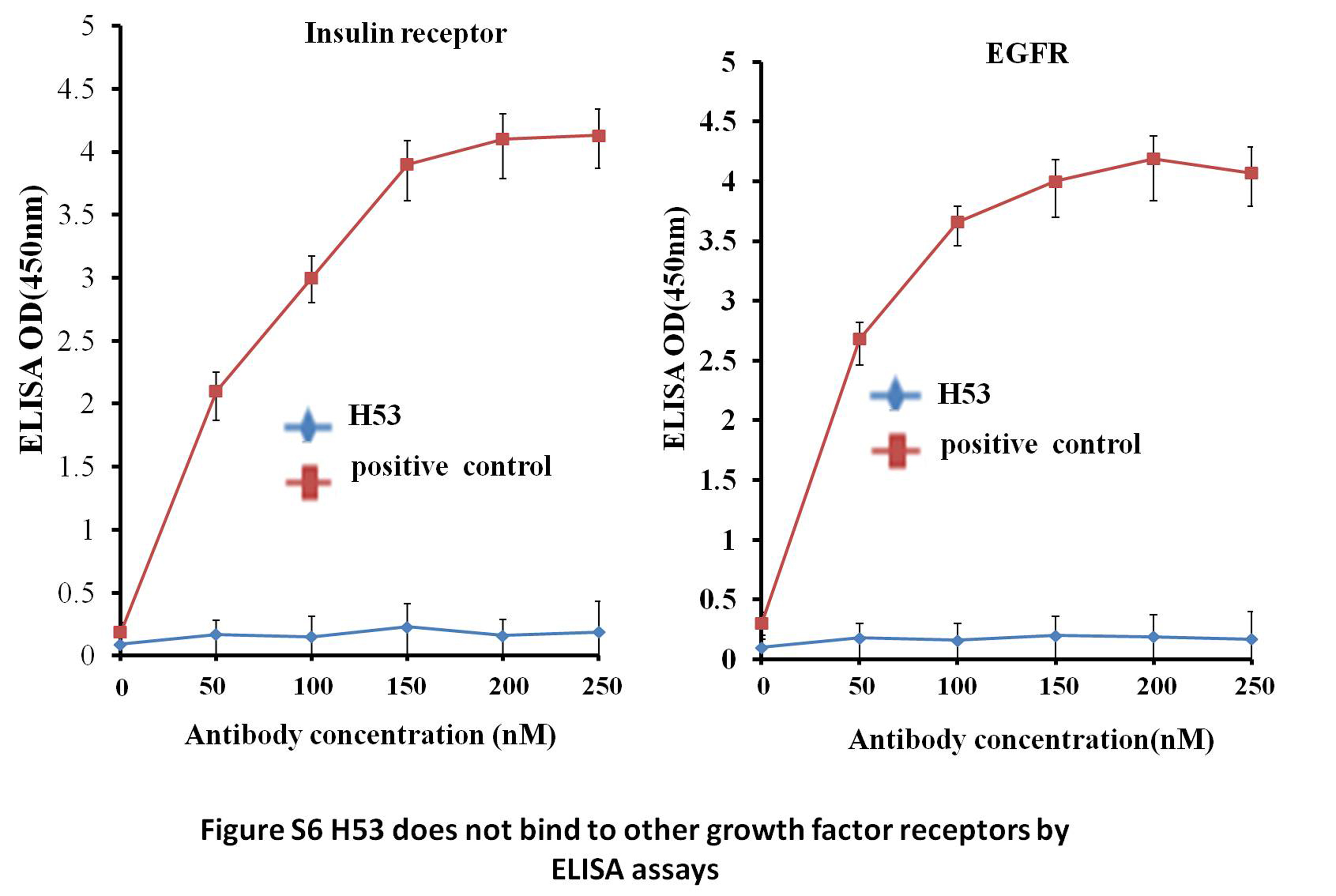

Supplement: Supplementary file 6 [file image6.jpeg]

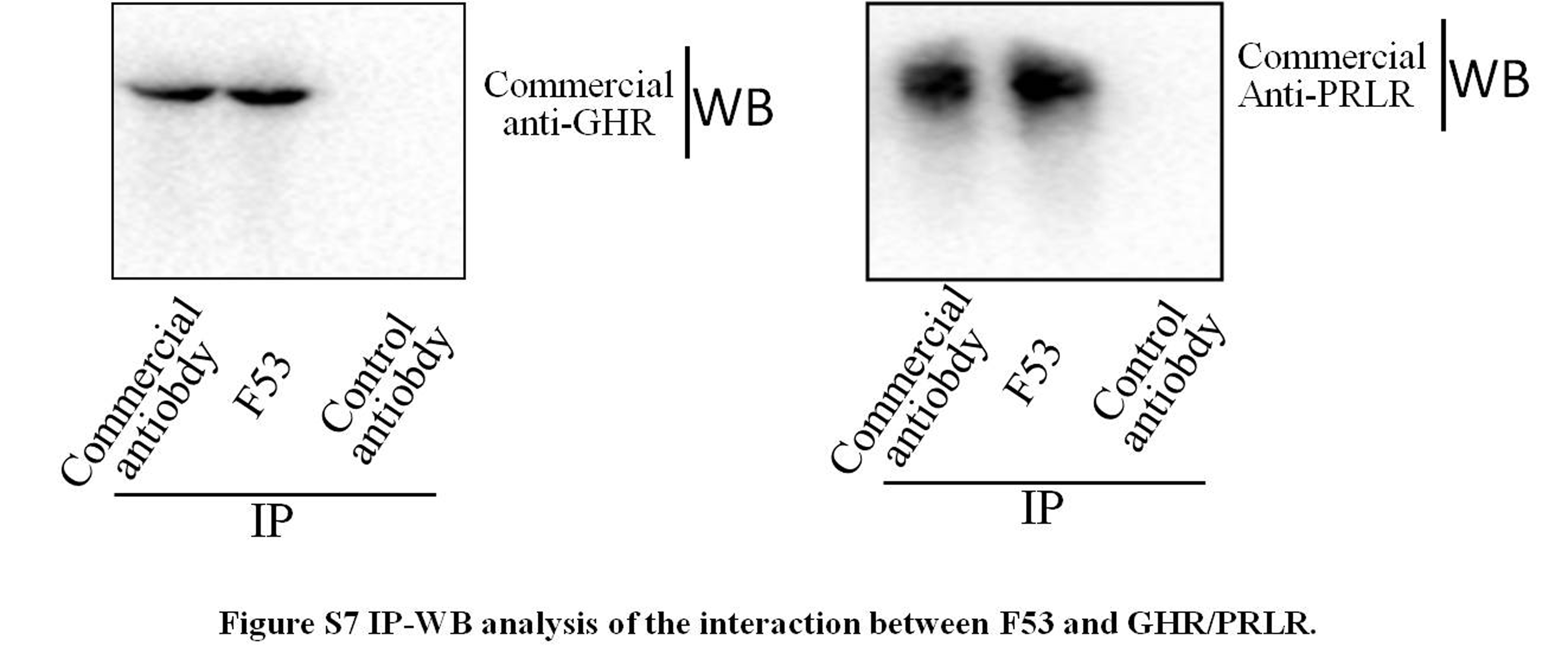

Supplement: Supplementary file 7 [file image7.jpeg]

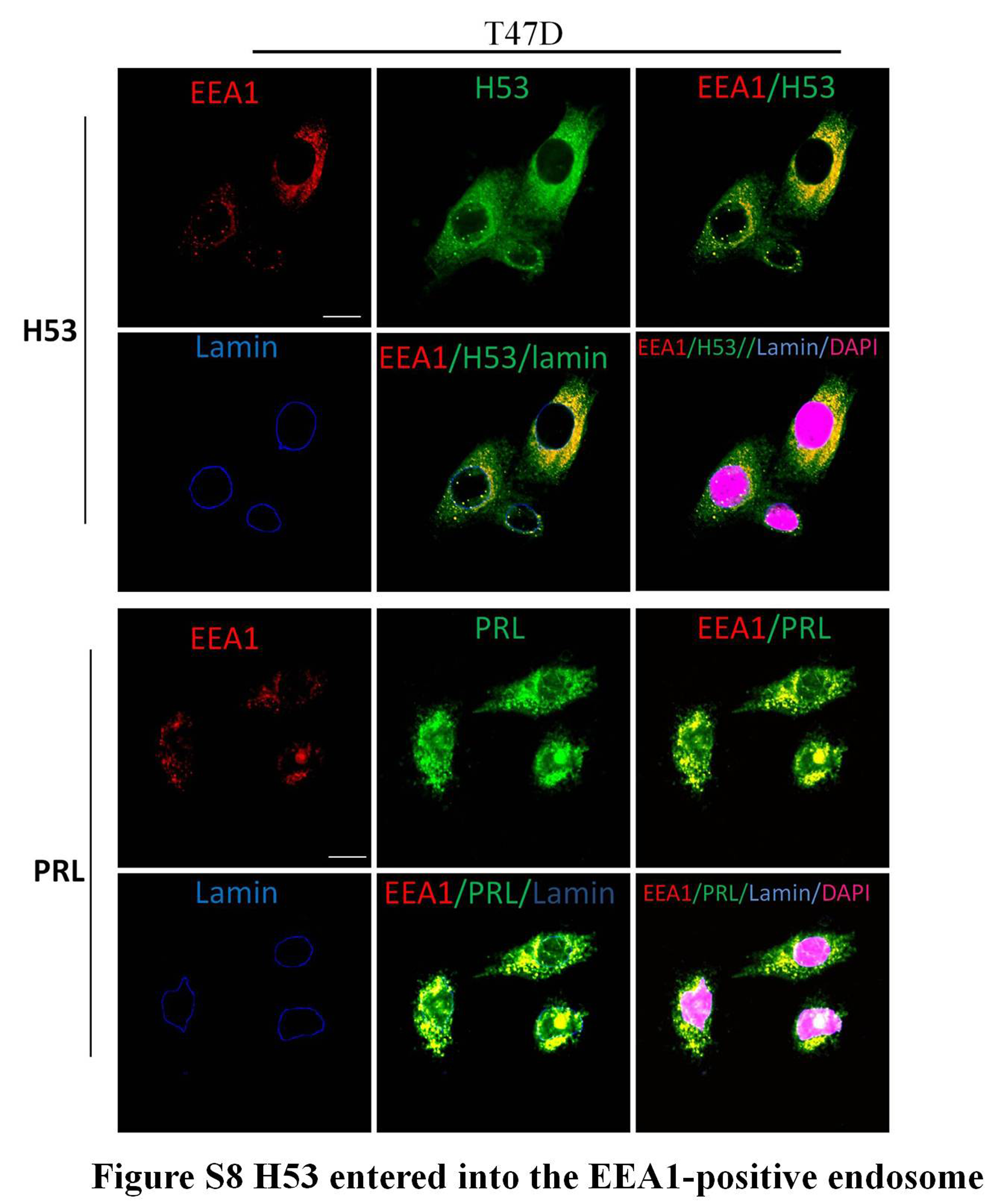

Supplement: Supplementary file 8 [file image8.jpeg]

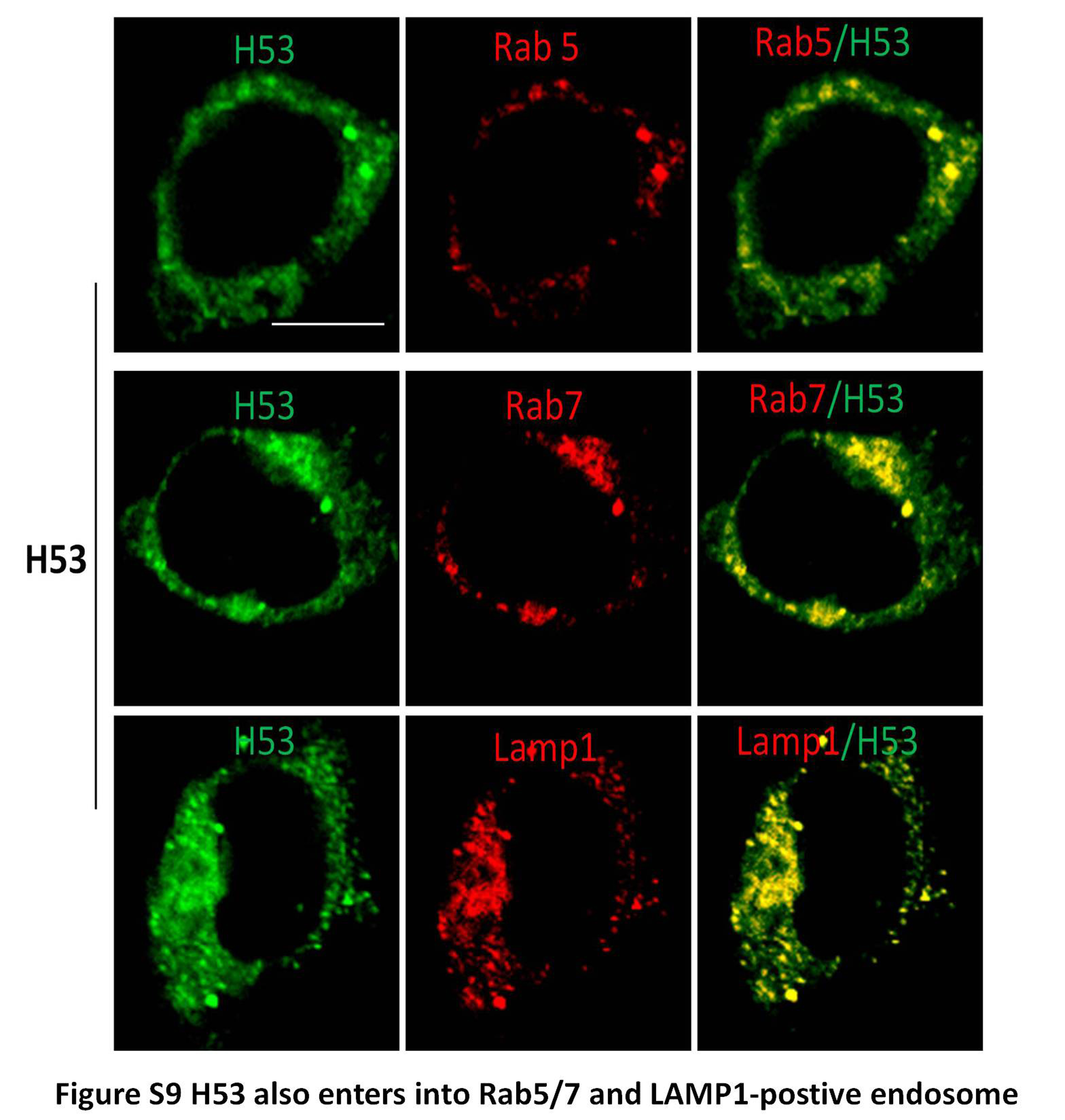

Supplement: Supplementary file 9 [file image9.jpeg]

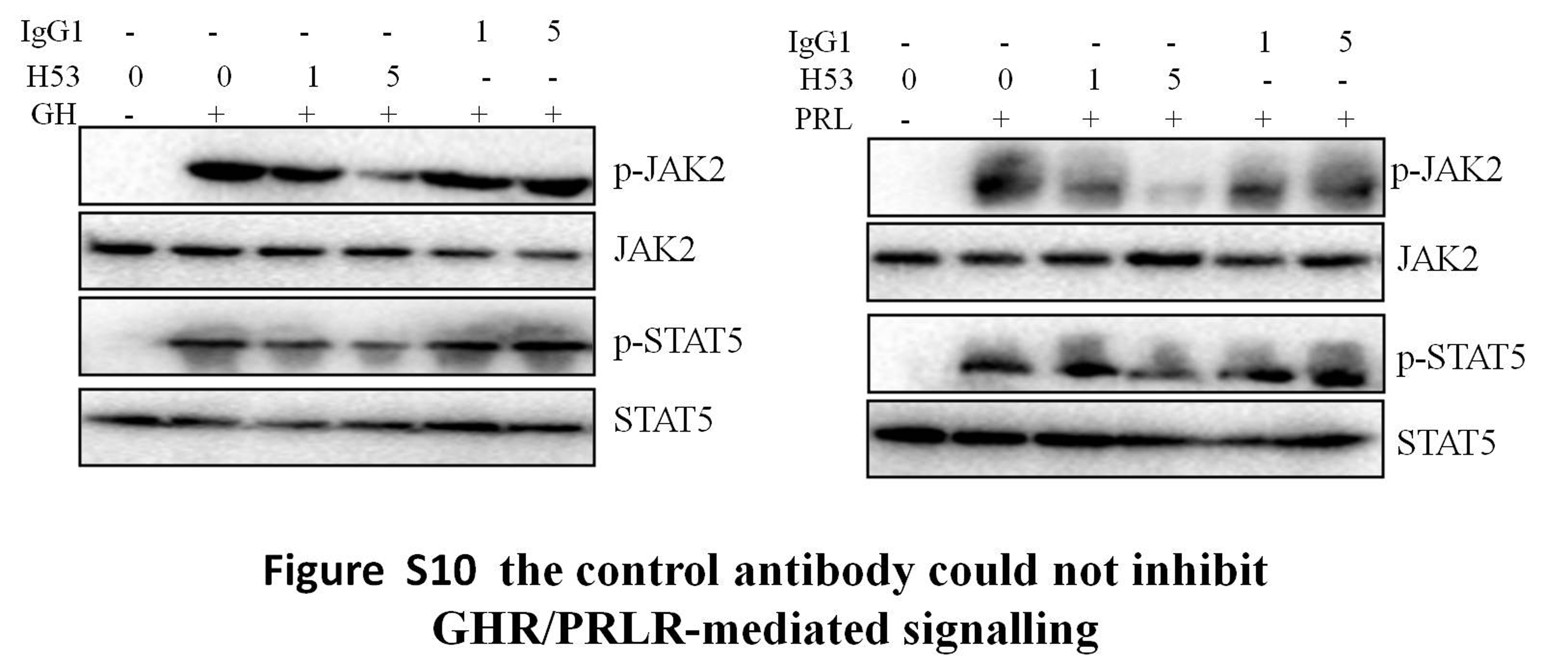

Supplement: Supplementary file 10 [file image10.jpeg]

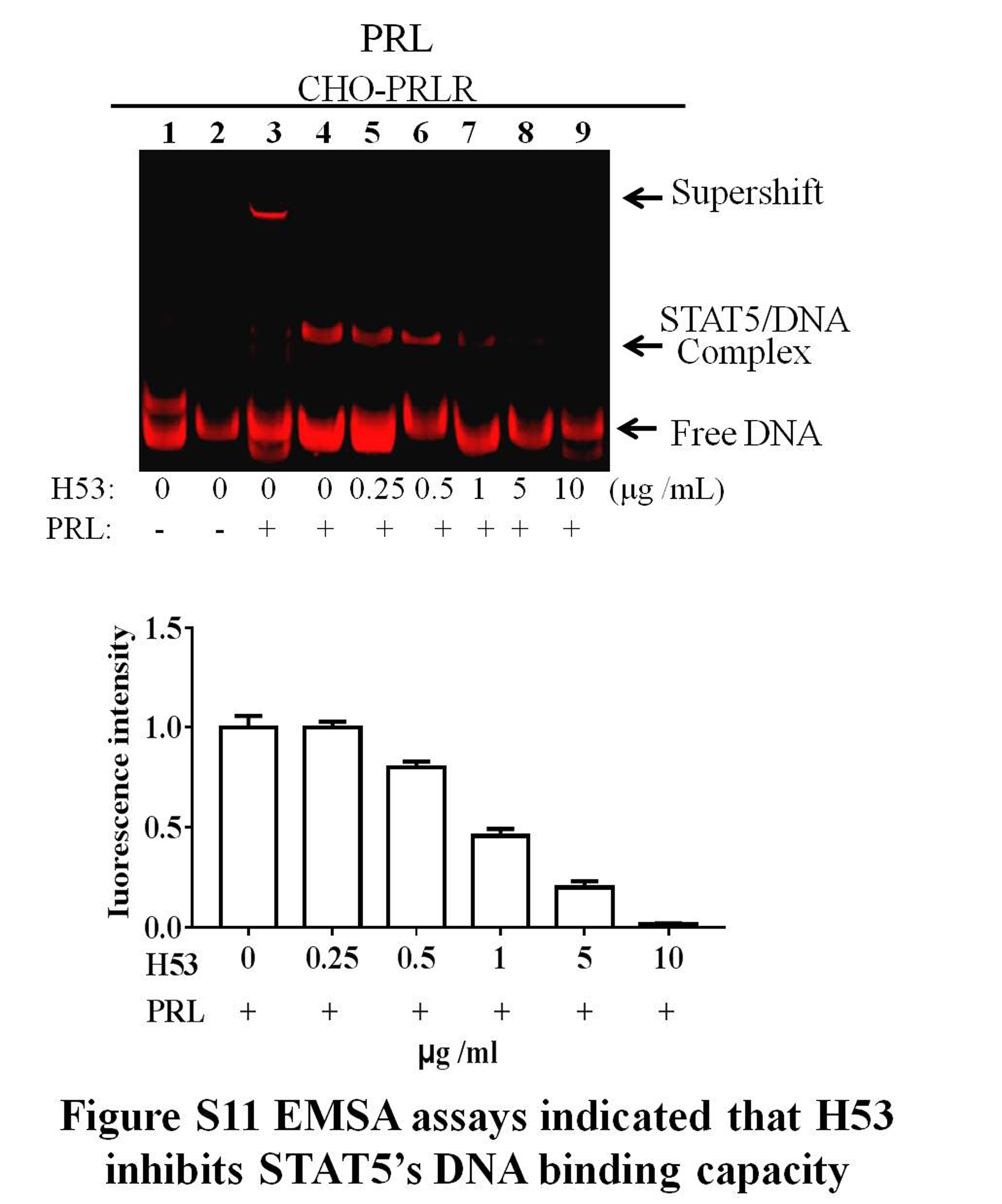

Supplement: Supplementary file 11 [file image11.jpeg]

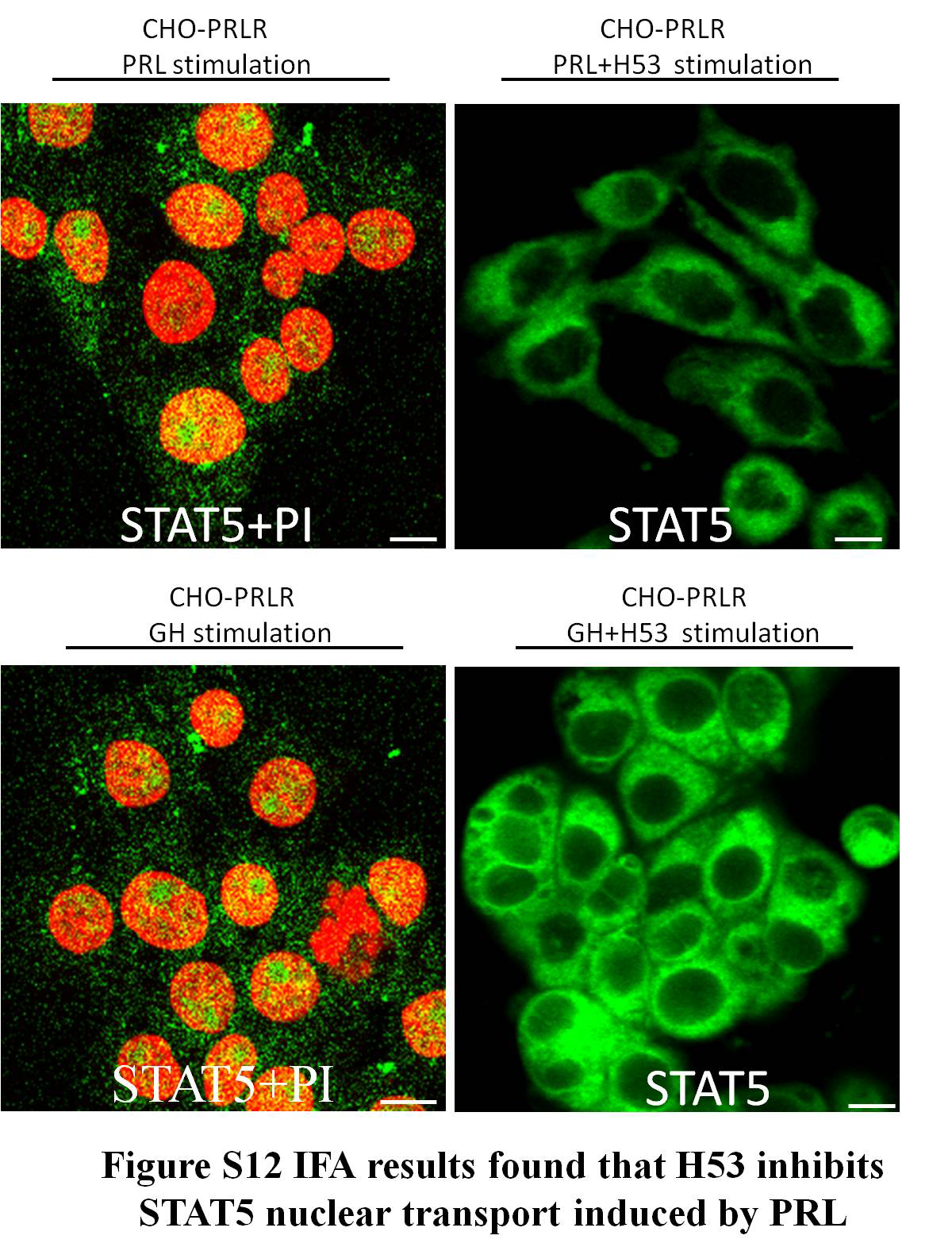

Supplement: Supplementary file 12 [file image12.tif]

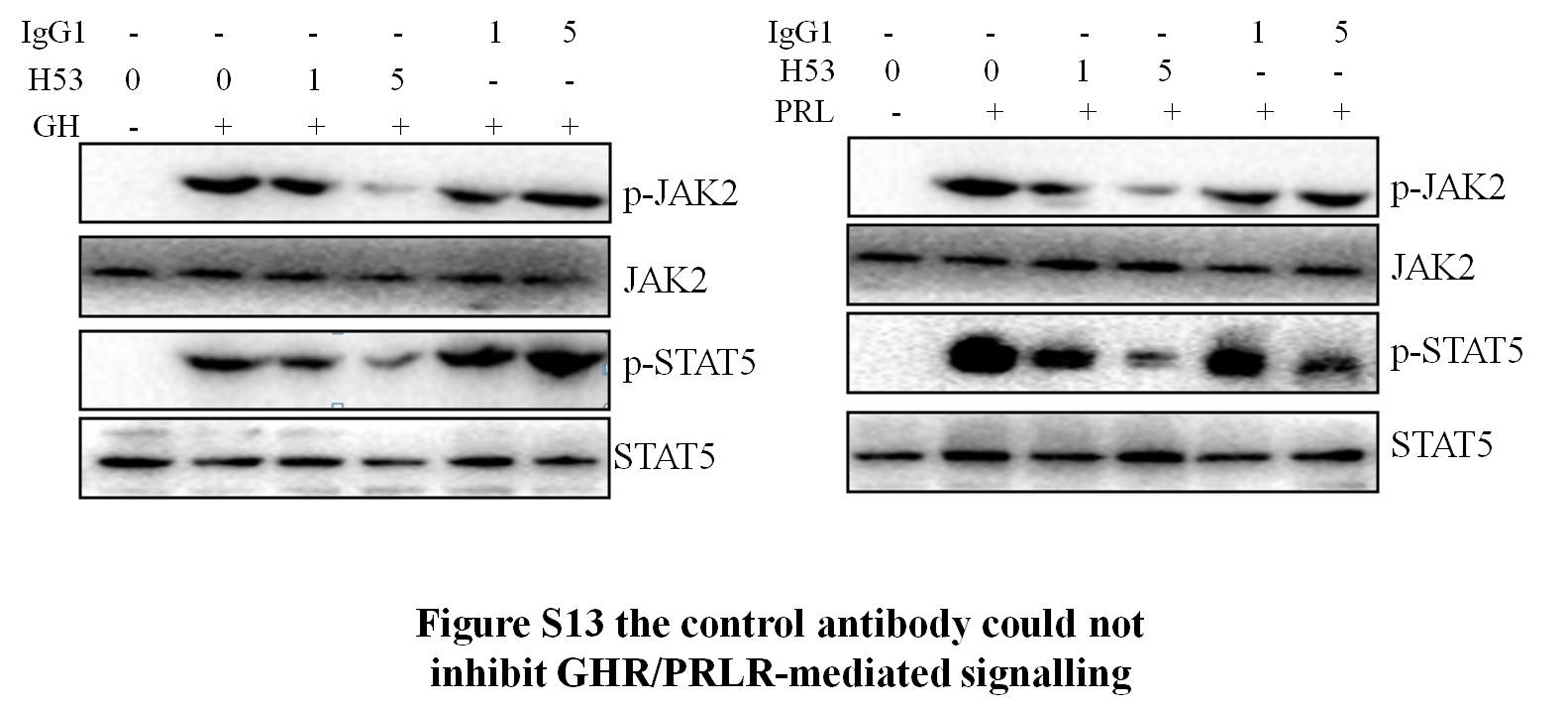

Supplement: Supplementary file 13 [file image13.jpeg]

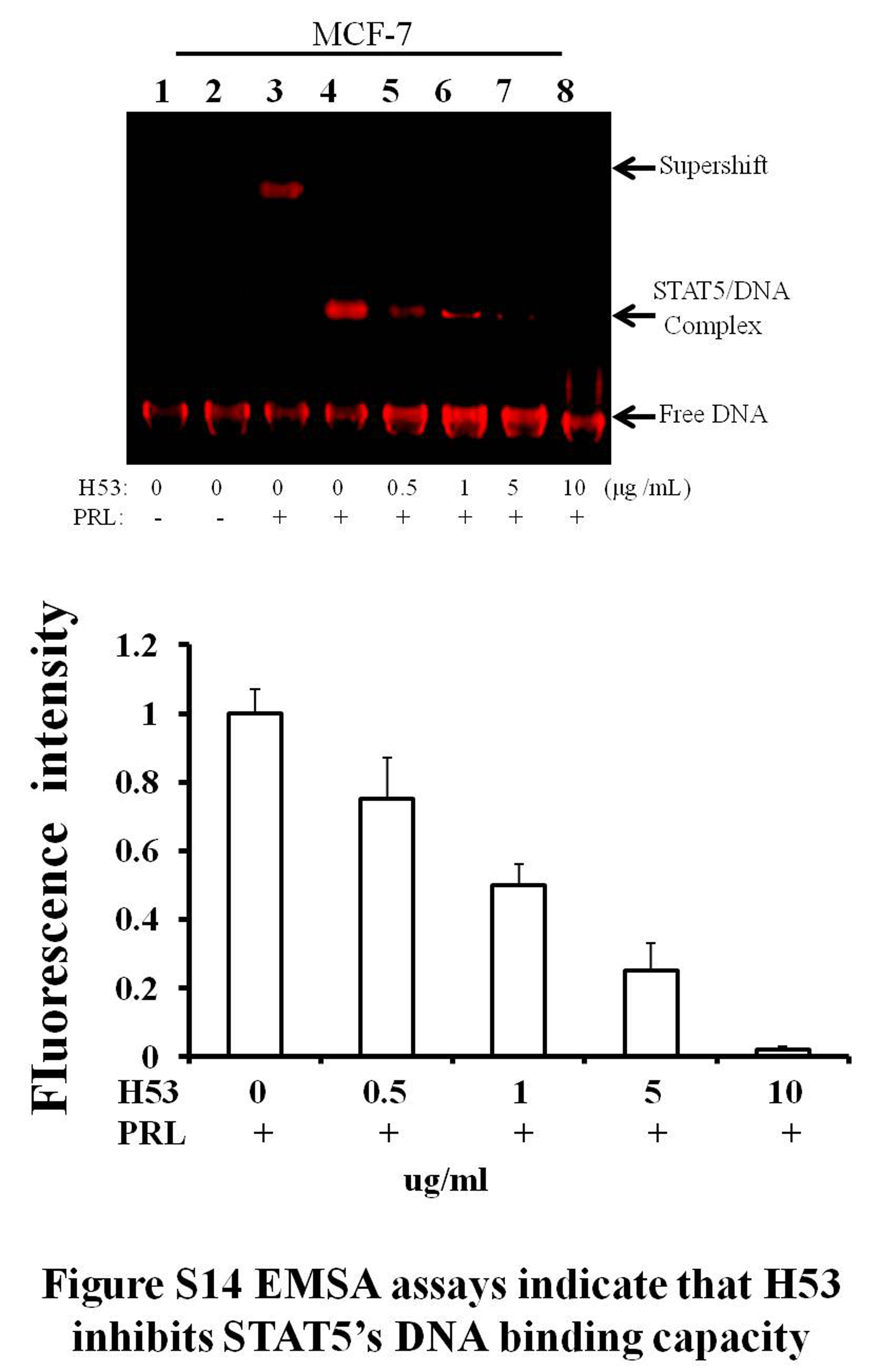

Supplement: Supplementary file 14 [file image14.jpeg]

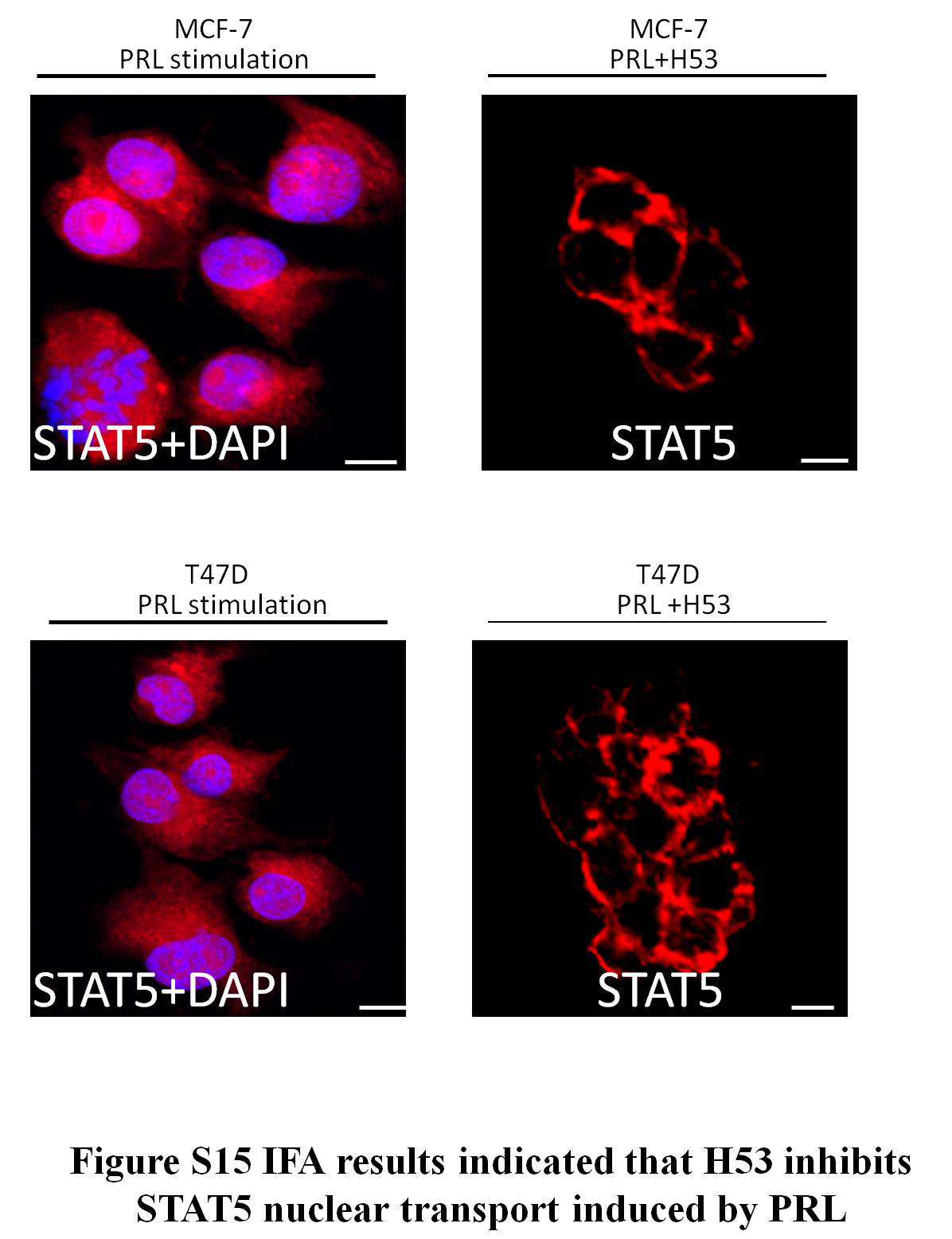

Supplement: Supplementary file 15 [file image15.tif]
